# Anti-HERV-K Drugs and Vaccines, Possible Therapies against Tumors

**DOI:** 10.3390/vaccines11040751

**Published:** 2023-03-28

**Authors:** Sepideh Hosseiniporgham, Leonardo Antonio Sechi

**Affiliations:** 1 Department of Biomedical Sciences, University of Sassari, 07100 Sassari, Italy; 2 Unit of Microbiology and Virology, Agency of University Hospital (AOU), 07100 Sassari, Italy

**Keywords:** HERV-K, tumorigenicity, cancer, immunogens, vaccine, antibody, melanoma, breast, ovary, prostate

## Abstract

The footprint of human endogenous retroviruses (HERV), specifically HERV-K, has been found in malignancies, such as melanoma, teratocarcinoma, osteosarcoma, breast cancer, lymphoma, and ovary and prostate cancers. HERV-K is characterized as the most biologically active HERV due to possession of open reading frames (ORF) for all Gag, Pol, and Env genes, which enables it to be more infective and obstructive towards specific cell lines and other exogenous viruses, respectively. Some factors might contribute to carcinogenicity and at least one of them has been recognized in various tumors, including overexpression/methylation of long interspersed nuclear element 1 (LINE-1), HERV-K Gag, and Env genes themselves plus their transcripts and protein products, and HERV-K reverse transcriptase (RT). Therapies effective for HERV-K-associated tumors mostly target invasive autoimmune responses or growth of tumors through suppression of HERV-K Gag or Env protein and RT. To design new therapeutic options, more studies are needed to better understand whether HERV-K and its products (Gag/Env transcripts and HERV-K proteins/RT) are the initiators of tumor formation or just the disorder’s developers. Accordingly, this review aims to present evidence that highlights the association between HERV-K and tumorigenicity and introduces some of the available or potential therapies against HERV-K-induced tumors.

## 1. Introduction

A family of dsDNA viruses [1] is called HERV, which was embedded [2] into the genome of primates almost 100 million years ago and vertically handed down to the following generations via germ-lines in a Mendelian pattern [3,4]. In fact, cleavage between old- and new-world monkeys led to incorporation of HERVs into the human genome around 35–45 million years ago [5]. As a result of consecutive replication and movement of transposons, multicopy and single-copy proviruses are scattered throughout the cells’ DNA [4], in which almost 8% of human DNA is constituted of the HERV DNA [6,7]. These so-called fossil viruses are the remainder of retroviruses that previously infected cells. HERVs genetically resemble the present species of exogenous retroviruses, such as human immunodeficiency virus (HIV) and human T cell leukemia virus (HTLV).

Retroelements are a group of repetitive DNA sequences that consist of 42-43% of transposon elements in the human genome [8], and they are classified into two main groups based on possession or lack of long terminal repeats (LTRs). LINEs and short interspersed nuclear elements (SINEs) belong to non-LTR retroelements consisting 33% of the human genome [9]. HERV and retrotransposons that are differentiated based on the existence or lack of Env gene belong to LTR retroelements (8% of the genome of humans) [10]. As with other retroviruses, Gag, Pol, and Env regions that are inserted between two LTRs play an important role in infectivity [4]. Expression of HERV genes is regulated by nucleotide sequence motifs located in LTRs [4], which comprise RNA regulatory sequences and transcription factor binding sites [7]. While capsid and envelope proteins are encoded by HERV Gag and Env genes, respectively, enzymatic products that contribute to replication, integration, and cleavage are expressed by the HERV Pol gene [4]. The current HERVs have been imperfected over millions of years of evolution compared to their ancestors. This is because the primary HERVs underwent frequent integrations and were exposed to some damage [7]. For instance, due to the selection phenomenon, the majority of pathogenic domains no longer exist in the current HERVs [11]. While mutations [12], deletions, converting the coding sequences to the terminal sequences [4,13,14,15,16], and epigenic control [12] contribute to inactivity or impairment in HERVs genes, specific physiological circumstances could bring these genes back into expression in some HERVs [4,12].

Based on the main divergences that occurred in the DNA sequences of animal retroviruses and HERVs, HERVs were grouped into three main classes [17]. Class I has similarities to infectious mammalian type C viruses and is distributed into six groups. The members of these groups are as follows: HERV-HF, HERV-RW, HERV-ERI, HERV-T, HERV-IP, and ERV-FRD. Among class 1 members, HERV-H, HERV-I, and HERV-R (ERV-9) have homologous sequences with murine leukemia virus (MuLV) and baboon endogenous virus (BaEV) in Pol, Gag, and Env regions. Class II has a vicinity with mammalian type B (i.e., MMTV) and type D retroviruses and includes 11 groups [18] of HERV-K (HML-1), HERV-K (HML-2), HERV-K (HML3), HERV-K (HML-4), HERV-K (HML-5), HERV-K (HML-6), HERV-K (HML-7), HERV-K (HML-8), HERV-K (HML-9), HERV-K (HML-10) [19], and HERV-K (HML-11); this multitude means that various infections occurred at the germ-line level [18]. The members of class II have lysin tRNA; this suggests that class II originated from type B and D retroviruses [20]. HERV-L is the only member of foamy HERVs discovered in class III. This class has homology with HTLV-I in the LTR zone [19]. Some HML-2 proviruses could particularly be found in humans and some others in both humans and chimpanzees. This proves that incorporation following human–chimpanzee divergence happened at the genome level, and this corresponds to the fact that HML-2 is the most complete and active provirus of its family [21]. HML-2 is distributed in the genome within 91 complete proviruses and 944 solitary LTRs, in which the LTRs resulted from unequal crossing over [18].

HERVs could affect biological procedures, such as placenta development, capability of stem cells in differentiation of various cell types, and teratocarcinoma cell lines migration and their viability [22,23,24,25,26,27,28]. The possible roles of HERVs in formation of cancers have been reported in several studies. There might be an association between competence of HERVs in coding viral-like particles and their potential in promoting carcinogenicity or inducing autoimmune diseases [4,13,14,15,16]. Among the HERVs-dependent activities that potentially encourage carcinogenicity, the following are of importance: increasing HERVs RNA expression [29], functional proteins expression [30], retroviral-like particle secretion [31], emerging new promotors [32], and activation of proto-oncogenes [33]. For instance, in real-time (RT) PCR-based analysis, tumorigenicity in liver, lung, testis, and colon has been associated with overexpression of HERV-K, HERV-H, and HERV-P envelope proteins (Env), respectively [34]. Further RNAScope ISH analyses of lung cancer cells and blood samples collected from both lung cancer and healthy individuals depicted that HERV-K Gag, Pol, and Env were highly transcribed in cells and blood of cancerous patients rather than in healthy individuals, in which higher transcription of HERV-K (HML-2) was associated with lung cancer severity. Therefore, the transcription levels of HERV-K Gag, Pol, and Env could be considered as indicators for detection of lung cancer [35].

HERV-K is the most active human endogenous retrovirus [36] that is categorized into two subtypes, 1 and 2, based on possession or lack of a sequence of 292 bp at the Pol–Env frontier [37]. Possession of these extra nucleotides enables type 2 to be spliced, whereas type 1 does not have this capability [38]. These subtypes consist of a total of 30 to 50 members that are extremely conserved in old-world monkeys and apes [36]. HERV-K provirus contains intact open reading frames (ORF) that enable it to induce production of viral particles [36]. However, the ORF for Gag, Pol, and Env in HERVs could prevent the cells from reinfection with other viruses and exogenous retroviruses. While capability of HERV-K, as in other HERVs, in generating horizontally transmittable [39] infectious retroviral particles might be restricted via post-insertional mutations, some HERV-Ks could retain their infectivity through insertion of ORFs necessary for coding essential viral proteins and conveyfrom one generation to another vertically [39,40].

Syncytin 1 and Syncytin 2 are, respectively, derivatives of HERV-W- and HERV-FRD-Env proteins that are involved in cell–cell amalgamation during evolution of the placenta in humans [41,42,43,44] and development of trophoblast [41]. Based on a study in mice, it is proposed that HERV Env proteins prevent host cells to be reinfected with exogenous retroviruses by blocking the cell’s surface receptors, and also a derivative protein of Gag restrains the host cell to be infected by other retroviruses [45]. HERV-K could only be expressed in specific cell lines, such as germ cell tumors, melanomas, or organs such as the placenta [46]. HERV-K sequences are highly expressed in teratocarcinoma cells [31,47] and code for teratocarcinoma-derived virus (HTDV; since they are recombinant of HTDV, these endogenous retroviruses are also called HERV-K/ HTDV) and HERV-K Gag [36]. The recent activity has been proven by immunoelectron microscopy analyses [36]. The lack of methylation in the teratocarcinoma cell line increases expression of HERV-K Gag protein in the involved cells [48]. In the meantime, the viability and migration of teratocarcinoma cells are guaranteed by HERV-K accessory protein Np9.

HERV-K has even been tracked in patients with leukemia, in which a high concentration of HML-2 Gag mRNA was estimated in PBMC of patients with leukemia rather than healthy individuals [49]. This is under the condition that blood samples taken from patients with chronic and acute myeloid leukemia, CML, and AML, respectively, contained high percentages of HML-2 Pol mRNA [50,51]. Recently, it has been suggested that HERV-K contributes to expansion of multiple myeloma (MM: plasma cell cancer that involves bone marrow and leads to crucial bone lesions, anemia, hypercalcemia, and kidney injury [52]), in which expression of HERV-K was detected reliably meaningfully in MM patients compared to monoclonal gammopathies of undermined significance (MGUS: precancer of clonal plasma that affects monoclonal immunoglobulin (M cells) in serum) [53,54]. This overexpression was aligned with restraint of *TP53* and *CDKN1 A,* tumor suppression genes [54].

On the other hand, several studies suggested that expression of HERV-K proviruses contributes to germ cell tumors (GCTs, i.e., ovary and testis), in which the titers of antibodies directed against HERV-K Gag or Env proteins were estimated at 62.5% or 80% in samples taken from patients with GCTs [55].

In recent years, a PCR-based target enrichment sequencing protocol for HERV-K (HML-2) (PTESHK) loci has been developed that could screen the samples and discriminate reference and non-reference HERV-K (HML-2) loci [56]. Execution of this method along with other novel approaches could disclose essential information regarding attribution of HERV-K (HML-2) in various cancers and autoimmune disorders [56].

Previous studies on the immune system, placental tissues, and early embryo pluripotent cells depicted that HERVs are potential therapeutic options as they are essential sources of regulatory genes [28,57,58,59,60]. While expression of HERVs could induce carcinogenicity or tumorigenicity, HERVs could be exploited to stimulate tumors and activate innate/adaptive immune responses (i.e., interferon responses) [61]. Until now, several HERV-K targets have been nominated to be used in vaccination against HERV-K-associated cancers and tumors. All activated vaccines and drugs against HERV-K have been proposed or designed based on their immunogenicity against Gag, Pol, or Env epitopes and other HERV-K protein products. These therapeutic options differ based on their mechanisms of action. The accessory Rec protein is an HERV-K gene product that can up-regulate androgen receptor and testicular zinc finger protein and affect gene expression in patients with prostate cancer [62]. In addition, HERV-K proteins can attach to specific RNAs and influence their translation [63].

**Figure 1:**
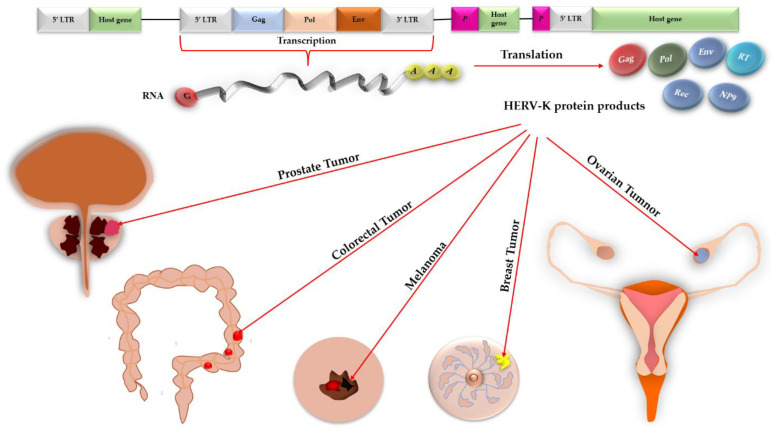
Expression of HERV-K in the genome of various host cells, production of HERV-K protein products, such as Gag, Pol, Env, NP9, Rec, and RT (reverse transcriptase), and the possible role of HERV-K protein products in tumorigenicity in different human organs.

Accordingly, this review aims to discern the tumorigenic roles of HERV-K and its products in various malignancies generated in the skin, teratoma, bone, and connective tissues, colon, breast, ovary, and prostate against these epitopes (Figure 1) and introduce some effective drugs/vaccines that are administrated against HERV-K-induced tumors.

## 2. Evidence of Immunogenicity against HERV-K Genes and Viral Particles in Patients with Tumors

Gag, Pol, and Env are three HERV genes that are essential for propagation of all retroviruses. As a result of expression of these genes in germ cell tumors (e.g., teratocarcinoma cell lines), three polyproteins are synthesized that could be converted into active structural or enzymatic subunits under viral or host protease exposure [20]. As the mutations randomly occur during replication of the host genome, HERV genes inevitably undergo mutations that reduce the chance of inheriting open reading frames (ORFs) for Gag, Pol, and Env genes to the progenies [20]. Some HERV sub-families, such as HERV-K, especially the HML-2 subtype [64], could retain intact [65] ORFs essential for coding all viral proteins [14,66], including Gag, Pol, and Env [20,67] plus small accessory Rec/Np9, in which the mentioned genes could be expressed from 5′-LTR [67] through alternative RNA splicing that enables a single gene to produce multiple proteins [67,68]. These proteins could be united as virus-like particles that lack genetic materials [67,69]. Analyses of the human genomic library under low-stringency conditions via probes isolated from human retroviruses led to discovery of deficient proviruses belonging to HERV-E, HERV-R, and HERV-HTDV/HERV-K families; among them, HERV-HTDV/HERV-K proviruses were debatably deficient and contained all viral genes for expression of central ORF, Gag, protease, polymerase, and Env proteins. Further experiments depicted that escalated levels of HERV-HTDV/HERV-K mRNA existed in teratocarcinoma cell lines and testicular tumors compared to normal tissue and organs, such as placenta [20].

Various studies demonstrated that HERV-K contributes to formation of some types of tumors in humans, in which the immune responses (antibody) induced against HERV-K were estimated higher in patients diagnosed with cancers than normal individuals [49].

### 2.1. HERV-K and Melanoma

Melanoma is one of the most lethal skin malignancies, and, if it is diagnosed in the initial phases, it will be managed not to invade and spread to other areas [70].

The titers of antibodies against HERV-K could be a marker in detection of patients with melanoma [71,72], in which higher titers of anti-HERV-K were estimated to be associated with decreasing lifespan in these patients [21].

LINE-1 is a group of non-long terminal repeats retrotransposons (retrotransposons are DNA products produced through reverse transcription that have the capability to relocate to a new region of the host genome in order to become incorporated into it [73]) that are distributed in the genome of many eukaryotic creators. In fact, LINE-1 contributes to insertion of DNA sequences conveyed by an RNA intermediate [74]. In normal conditions, expression of LINE-1 is restrained to avoid DNA impairments. However, activation of LINE-1 could lead to some types of malignancy [74]. A study depicted that methylation of LINE-1 and HERV-K are diagnostic biomarkers of melanoma tumors, with both decreasing in the clinical stages of the disease, whereas hypomethylation of HERV-K, but not LINE-1, could happen in the chronic stage of melanoma [75].

Up to now, HERV-K retroviral particles, such as Gag, Pol, Env, and Rec proteins, have been identified [76] in melanomas, metastases, and melanoma cell lines through immunohistochemistry, immunofluorescence, and Western blot analyses [77]. Exposure of Madin–Darby bovine kidney (MDBK) cells to the particles released by melanoma cells led to induction of infection to the MDBK cell [76]. Although these particles could not infect the cultured melanocytes due to the fact that retroviruses only target cells engaged in proliferation and the recent step lacks speed in monocytes, any de novo insertions by either retrotransposition or infection could comparably encourage expression of cellular genes and have the potential to impair ORF and tumor suppressor genes that could, respectively, influence expression or function of various genes and activate the genes that take part in carcinogenesis [76]. In a transcriptional analysis of cDNA sequences extracted from melanoma samples, almost 23 loci of HERV-K (HML-2) were characterized [78], determining that the proportion of these loci varied by the sample type [78]. For instance, one locus was just found in a melanoma sample but not in melanocytes; some loci contained ORF for coding Gag or Env proteins; Env, Rec, and Np9 could only be expressed in melanoma but not melanocytes; UVB radiation influenced the loci properties, reducing their transcription capability in both melanoma and melanocytes [78].

HERV-K Env and Rec are differed genotypically and phenotypically [79]. While Np9 and Rec are resulted from Env splicing, excessive expression of Env and Rec can differently influence the degree of malignancy in melanoma [79]. In addition, up-regulation of Env could enhance invasibility of tumors via activation of ERK1/2, and overexpression of Rec hampers epithelial–mesenchymal transition (EMT) and melanoma metastasis [79]. This might be due to the capability of Rec protein in binding to the zinc-finger protein [80] or glutamine-rich tetratricopeptide repeat protein (HSGT) plus suppressing the transcriptional factors [62,81]. On the other hand, melanocyte-inducing transcription factor (MITF) was found embedded in long terminal repeats (LTR-Hs) elements, affecting proliferative melanoma, in which any impairments in MITF could deteriorate expression of HERV-K [79]. Furthermore, imperfections in Rec in proliferative melanoma cells could reduce production of MITF mRNA and over-expression of EMT, which both can impose invasibility on proliferative melanoma cells [79] (Table 1).

### 2.2. HERV-K and Teratocarcinoma

Teratocarcinomas include mixed germ-line tumors that could involve a variety of tissues, including male and female sexual gonads [82], placenta, extraembryonic membranes, and umbilical cord [82,83].

HERV-K particles were detected while liberating from teratocarcinoma cell lines [46]. Even though HERV-K viruses have never been isolated from or cultivated in placenta in vitro, production of the retroviral particles was reported in induced teratocarcinoma [106]. It seems that HERV-K codes HTDV [13] (or HTDV/HERV-K) [47]. This has been proven through immunofluorescence analysis via antiserum containing anti-HERV-K recombinant Gag protein on teratocarcinoma cell lines that highly produced HTDV, depicting that specific reactivity existed between anti-HERV-Gag and HTDV particles. Further Western blot analysis revealed that the antiserum identified a 30 kDa protein corresponding to the HTDV central protein [13]. Interestingly, Northern hybridization of different members of HERV-K cloned from teratocarcinoma genomic DNA along with PCR and RNase protection assays confirmed that various HERV-K proviruses could be presented in a single teratocarcinoma cell line [47,107]. Comparison between expression pattern and morphological characteristics of HTDV/HERV-K proviruses extracted from different teratocarcinoma cell lines depicted that the retroviral particles in these cell lines differed by type of viral surface proteins and free mature virions, suggesting expression of different HERV-K proviruses in teratocarcinoma cell lines [47].

A recombinant HERV-K transmembrane envelope (TM) protein and an immunosuppressive peptide existing in RM protein were detected among teratoma-liberated viral proteins, contributing to alteration in expression of several cytokines and cellular genes. This functionality of TM protein in HERV-K is similar to TM in HIV-1 [46]. HERV-K (HML-2) was detected in both teratocarcinoma cell lines of GH and Tera-1; however, GH released more numbers of HML-2 (4–5 times) than Tera-1 [84]. Precisely, the presence of HERV-K108 Env was also proven in HML-2 isolated from Tera-1 by cDNA sequencing [84]. Based on density gradient analysis, the lighter fractions (1.16 g/cm^3^) contained more HML-2 than other fractions [84]. Surface (SU) and TM subunits were almost glycosylated [84]. This is under the condition that variant transmembrane envelope proteins (P24-TM) were detected and shortened to 24 KDa resulting from deglycosylation of HML-2 GH and Tera-1, in which this truncation probably happened after translation at the C-terminus of TM [84]. Furthermore, a drop in the amount of SU and activity of RT could be seen after deglycosylation [84]. HML-2 in the frame of particles or assembled virions was trapped and transferred by microvesicles (MVs) [84], in which HML-2, in general, can induce immune responses in the body [108], and its TM protein could only promote delivery of cytokines, such as IL-6 and IL-10 [46] (Table 1).

### 2.3. HERV-K and Osteosarcoma

One of the most typical cancers that appear in bones [109] is called osteosarcoma (OS). This cancer initially involves the long bones; however, it might progress to other bones as well [110].

Improper function of transposable elements (TE) could lead to a variety of tumors. The footprint of this dysfunctionality was found in OS by RNA and whole genome sequencing and methylation data analyses [85]. Meaningful overexpression of LINE-1 and Alu, SVA, and HERV-K, along with an increase in DNA repair responses, were reported in OS cases [85]. Transposons impose damages to DNA, and severity of DNA repair responses depends on the number of broken ends that resulted from TE insertions [111,112]. In fact, expression and translocation of transposons are the product of a three-step biological process including methylation of cytosine residue in DNA at the CpG region following a chromatin transformation step that makes it inaccessible to transcription factors; activation of piwi protein and piwi-relevant RNA complexes in order to impede transcription of repeated elements and eliminate transcribed retrotransposon RNAs; and P53 regulating stress signals in response to the broken double-strand DNA and insertion of retrotransposons following cell cycle arrest, aging, or apoptosis that leads to DNA repair or programmed cell death [112,113]. Over-expression of LINE-1 could also result in LINE-1 insertions and tumorigenicity. To proliferate LINE-1 retrotransposons, RNA sequences should undergo reverse transcription to be converted into cDNA and embedded into a new region in the genome. However, overexpression of LINE-1 might enhance the rate of LINE-1 insertion into adjacent genes that cause transcriptome interruption [111]. Moreover, LINE-1 transposons have the capability to translocate the genes of mutations and take part in epigenetic dysregulation [111]. Over-expression of LINE-1 was reported in OS [85] as well as invasive breast, head, and neck malignancies [114]. In a comparative analysis, 39599 and 17598 TE elements were recognized in OS patients and healthy controls, respectively, among which LINE-1 and HERV-K, respectively, had the highest and lowest proportions of overexpression in both samples taken from OS and healthy groups [85]. This is under the condition that the expression of both was higher in OS patients rather than in healthy controls [85] (Table 1).

### 2.4. HERV-K and Colorectal Tumors

Colorectal cancer (CRC) is the fourth [115] leading life-wasting malignancy that is frequently reported in Western countries [116]. The factors that enhance risk of CRC include aging, background health condition, and lifestyle [116,117]. Although bacteria such as *Fusobacterium* spp., *Bacteroides fragilis*, and enteropathogenic *Escherichia coli* might trigger cancer, any mutations in oncogenes, tumor inhibitor genes, and DNA repair operation induced by other agents could be associated with CRC [116].

RT PCR analysis of peripheral blood mononuclear cells (PBMCs) or solid tissue samples taken from colon cancer patients demonstrated that the pattern of HERV-K expression was different in human cells, in which malignant cells in the studied patients contained profuse amount of HERV-K Env transcripts class II (this class has lower homology of 71% with HERV-K 10), whereas low and non-malignant cells were diagnosed in a small quantity of both HERV-K Env class I (this class has higher homology of 96% with HERV-K 10) and class II [88].

HERV-K Env modulates the NUPR-Rb pathway in colon cancer. NUPR1 is a nuclear protein that was primarily diagnosed in pancreatic cancer patients [118]. This protein is importantly expressed in cancerous cells in response to stress and contributes to cell-cycle regulation [119,120], stress-associated apoptosis [119,120,121], production of reactive oxygen species (ROS) [122], DNA repair responses [123], and expansion and spread of tumor cells [121]. Interestingly, stress condition turns NUPR1 into a chromatin protein [86], in which it can attach to DNA and regulate dictation of specific genes [124], similar to other chromatin proteins [125,126]. RNA sequencing analysis depicted that removal of HERV-K Env via CRISPR-cas9 system could also decrease expression of NUPR1 in DLD-1, a colorectal adenocarcinoma cell line, and that was along with regression in level of ROS [124]. However, transfection of HERV-K Env-knock-out cells with HERV-K overexpression vector did not increase NUPR1 and ROS levels [122].

Previous studies depicted that a meaningful association could be expected between the lower methylation rate of LINE-1 or HERV LTR and some cases of tumorigenicity [127,128,129,130,131,132,133,134], in which the higher methylation level of them could be considered as a tumor-suppressing tactic in healthy cells [115,135]. Pyrosequencing analysis of samples collected from patients with advanced colon carcinoma revealed that methylation rates of LINE-1 and HERV-K were subsequently and significantly lower in tumor cells than normal tissues [115]. However, HERV-K Env and Pol were insignificantly transcribed within cancerous tissues and their normal counterparts. This is under the condition that the expression pattern of these proteins remarkably varied by the state of malignancy, in which the HERV-K Pol protein that has various isoforms due to proteolytic cleavage [136] was detected with a higher incidence in normal tissues around the tumors than tumors, whereas HERV-K Env protein was only manifested in tumor tissue [115]. At first sight, it might be comprehended that the higher expression of HERV-K Pol protein is just equal to production of higher RT; however, this up-regulation might be the initial sign of the conversion of normal cells into cancerous ones, resulting in reverse transcription of HERV-K RNA and incorporation of the cDNA to the genome of normal cells via HERV-K integrase [115]. Conversely, the elevated level of HERV-K Env protein in tumor areas might be associated with growth, invasiveness, and dissemination of tumors [115], in which CRISPR-cas9 analysis depicted that down-regulation of HERV-K Env has negative impacts on these processes [122].

Extracellular vesicles (EVs) play critical roles in tumorigenicity by contributing to filtration, metastasis, and resistance to immune responses [87]. Accordingly, it might be estimated that HERVs-captured EVs could influence innate immune responses in cancerous areas. To examine this hypothesis, two human colorectal cancer cell lines (metastatic and non-metastatic) were thoroughly hypomethylated using decitabine and excited to produce HERV [87]. Although LINE-1 and HERV LTR were hypomethylated in both cell lines, the methylation rate of HERV LTR was higher in all cancerous cells. Then, the induced cell lines were injected into zebrafish and the concentration of HERVs-positive EVs and their impression on innate immunity were evaluated accordingly [87]. The result showed that the incidence of HERV-K-captured EVs was remarkably higher than other HERVs-positive EVs in cancerous cell lines and this augmentation was aligned with a drop in the proportion of myeloperoxidase (*mpx*) and inflammatory cytokines, such as *IL1-β*, suggesting that HERV-K-positive EVs could regulate immune responses in tumor tissue [87] (Table 1).

### 2.5. HERV-K and Breast Tumors

One of the most common and silent-progressive cancers among women is breast cancer. It might have different manifestations, from formation of breast lumps and change in breast shape or size to release of fluid from nipples [89]. The tumors are almost responsive to treatment (70%–80%) if they are diagnosed at the early stages of the disease [137].

Presence of HML-2 Env mRNA has been reported in cases with breast tumors frequently [38,91]. Even expression of HERV-K Env in non-tumorigenic human breast cell lines led to modification of various cell types that existed in this area, from epithelial to mesenchymal following expansion of invasive tumors and metastasis [90]. Further microarray analysis for finding the source of this transformation demonstrated that HERV-K Env influences production of transcriptional factors, such as ETV4, ETV5, and EGR1, that contribute to activation of Ras-dependent extracellular-signal-regulated kinase (ERK)1/2 mitogen-activated protein (MAP) kinase pathway and its transformation [90]. T47-D belongs to the human mammary carcinoma cell lines that can liberate type 2 HERV-K that could be spliced to sub-genomic transcripts [38]. Further analysis depicted that treatment of T47D cells with estrogens and progesterone could enhance production of HERV-K reverse transcriptase protein (HERV-K-T47D-RT). HERV-K-T47D-RT might be used as an indicator of breast cancers at the early stages of the disease since, in a study, it was induced by 26% of tumor tissue and 18% of normal vicinal tissues taken from patients with breast tumors [138]. Similarly, significant growth in the number of HERV-K was also noticed in the MCF-7 breast cancer cell line that was treated with estrogen and Adriamycin [139].

In a comparative study, expression of four different loci of HERV-K was evaluated in four breast cancer types, including basal, Her2E, LumA, and LumB tumors [96]. The result revealed that HERV-K and specifically the Env gene could be expressed in basal tumors subtypes IDC rather than the other types of breast tumors [96], in which analysis of HERV Env cDNA extracted from four types of breast tumors depicted that almost 97% of the Env cDNAs belonged to type I HERV-K102 [140]. The specificity and immunomodulatory profiles of HERV-K make it a candidate for production of vaccines effective for breast tumors [96].

RT-PCR analysis and nucleic-acid-sequence-based amplification estimated high concentration of reverse transcriptase and HML-2 RNA in plasma samples taken from lymphoma and breast cancer patients containing the transcripts of Gag and Env genes [91]. Regardless of whether HERV-K (HML-2) could induce infectivity in lymphoma and breast cancer or not, HERV-K could be considered as a biomarker in diagnosis of tumors (i.e., the discrepancy between the healthy controls and lymphoma cases was around 8 folds copies/mL) [91], specifically at the early stages of breast cancer that the load of HERV-K mRNA and antibodies directed against HERV-K Rec or Env or N9 are remarkably higher than healthy individuals [141], although antigenicity of HERV-K N9 is lower than HERV-K Rec and Env due to its small size [141]. Serological analysis of samples taken from 119 patients with breast cancer (BC) and 76 healthy individuals demonstrated that the concentration of IgG directed against HERV-K Env was meaningful in 88% of BC patients but not in normal individuals. Further analysis of the recent cohorts unveiled activation of autologous dendritic cells pulsed with HERV-K env SU antigens in peripheral blood mononuclear cells (PBMC) [142].

Surprisingly, a relationship might exist among production of HERV-K Env protein, stage of the malignancy, and degree of lymph node metastasis in patients with breast tumors [143,144]. A study in China demonstrated that HERV-K Env positivity could be a predictor of the size of a tumor, TNM stage, and lymph node metastases in patients [143]. The correlations between each TNM stage (size, region, and distribution of tumor [145]), P53 mutation (mutation in TP53 gene leads to inactivation of P53 tumor repressor [146]), and nodal status with shorter longevity are meaningful in HERV-K-positive individuals [143] (Table 1).

### 2.6. HERV-K and Ovarian Cancer

No age limit has been determined for ovarian cancer; however, women at above 50 years old are categorized among the high-risk cohort. The patients first have inexplicable pelvic and abdominal signs [92]. Some diagnostic tests, such as transvaginal ultrasonography and serological cancer tests (Antigen 125), might disclose the malignancy in some cases but not in all individuals [92].

HERV-K attacks could also be recognized in patients with ovarian cancer, in which the presence of HERV-K transcripts, Env protein, and active reverse transcriptase was reported in ovarian tissues rather than healthy and non-invasive cancerous tissues [93]. Serological analysis of plasma in patients with ovarian cancer depicted that the levels of active reverse transcriptase and immunoreactive antibodies directed against HERV-K were noticeable in these cases, in which in vitro study depicted that those dendritic cells derived from the same origin but loaded with HERV-K Env protein could induce immunity by production of T cells, INFγ, and HERV-K-specific cytotoxic T lymphocytes (CTL) [93] (Table 1).

### 2.7. HERV-K and Prostate Cancer

Most tumors generated in prostate glands are non-aggressive; however, prostate cancer is a male concern in more than 50% of countries. Of delayed symptoms of prostate cancer, fatigue, anemia, bone pain, paralysis resulting from spinal metastases, and renal failure due to bilateral obstruction are more common [94].

Evidence showed that males suffering from prostate cancer had higher levels of HERV-K Gag RNA and its protein in their prostatic epithelial cell lines (specifically LNCaP) than non-cancerous cells and even healthy individuals (*p* < 0.001 for both), among which, in a study, almost 85% out of 27 prostatic cancerous cases had higher expression of HERV-K Gag RNA rather than HERV-K Gag protein (66.7%) [101]. It has been reported that overexpression of HERV-K Gag could strongly enhance expression of other HERV biomarkers but not HERV-K Np9. In contrast, expression of HERV-K Np9 had a remarkable impact on regulation of all tested transcripts except HERV-K Gag [101]. In fact, the titers of antibodies against HERV-K Gag could be measured in detection of patients with elevated risk of cancers and tumors, such as prostate cancer [67,147].

HERV-K Gag-related antigen, *NGO-Pr-54* mRNA, has also been detected in autologous patient serum through serological recombinant cDNA expression cloning (SEREX). *NGO-Pr-54* covers an intact ORF that encodes a 715-aa protein and has a similar size as Gag precursor; however, *NGO-Pr-54* at its full length could not be transfected into 239T cells (epithelial-like cells or human embryonic cells derived from the kidney [148]) [95]. This is under the condition that *NGO-Pr-54,* whose 5′ region was deleted, ZH042, could generate a 50 kDa protein through transfection, the same size as expected after translation of the Gag region [95]. Up to now, no definite reason has been found describing why the deletion of the 5′ region has an opposing effect on expression of the *NGO-Pr-54* region [95]. This might be due to the tendency of HERV-K to quickly react to stress, discrepancies, and environmental modifications, providing HERV-K with the capability of storing both partially spliced and fully developed mRNAs rather than in normal conditions [95,149]. In addition, export of HERV-K mRNA depends on presence of Rec proteins [150,151], and a lack of this protein due to deletion would hamper export of HERV-K mRNA. Further immunofluorescence analysis revealed that *NGO-Pr-54* protein is expressed on the surface of the cell and cytoplasm. Serological analysis depicted that the expression of *NGO-Pr-54* was obscure in normal prostate. In contrast, the greatest antibody reactivity against *NGO-Pr-54* was observed in patients with melanoma (13.2%) rather than cases with ovarian (5.6%), bladder (5.1%), prostate (4.2%), liver (4.1%), and lung (3.4%) cancers [95].

Additionally, one of the HERV-K Gag proteins is encoded by chromosome 22q11.23, which could be expressed in prostate cancer tissue [147]. Expression of this protein in prostate tissue is regulated by either removal of a methyl group from the promotor or induction via androgen. The level of antibodies directed against this HERV-K Gag protein was estimated at 6.8% in patients with prostatic cancer rather than in normal individuals (1.8%) via serological assessments. This analysis depicted that the amount of HERV-K Gag chromosome 22q11.23 expression and autoantibodies against this antigen enhanced by progression of prostate cancer in these patients [147].

In a study, quantitative reverse transcription-polymerase chain reaction (PCR) and pyrosequencing analyses of respective prostate cancer tissues and cell lines demonstrated that hypomethylation of retroelements might be associated with prostate cancer, generalized overexpression of HERV-K_22q11.23, and accessory Np9 transcript in some tumors; meanwhile, it was opposed to both DNA methylation in LTR and expression of HERV-K17 in cancerous tissues [97]. Application of androgen-responsive prostate cancer cell lines and steroid-hormone-responsive elements embedded in LTR segments could restrain expression of both HERV-K proviruses. This result highlighted that prostate cancer might be triggered by overexpression of specific subtypes of HERV-K [97]. Expression of members of the HERV-K family might follow a tissue-specific pattern. For instance, some HERV-K proviruses could only be expressed in prostate cell lines but not embryonal carcinoma cell lines (e.g., HERV-K17), and some could be expressed in both cell lines (e.g., HERV-K_22q11.23), and others could only be expressed in embryonal carcinoma cell lines (e.g., HERV-K_11q23.3 and HERV-K_22q11.21).

As with leukemia [49] and breast [142] cancers, a higher incidence of HERV-K Gag mRNA in PBMC was reported in samples taken from patients with prostate cancer, specifically smokers, than the healthy individuals [98]. According to this report, elevated level of interferon-γ was the other consequence of expression of HERV-K Gag in these patients. Age and ethnicity intervened in the intensity of HERV-K Gag mRNA and Env production, respectively [98]. While prostatic patients of all ages had higher levels of HERV-K Gag mRNA than healthy cases, overexpression of this mRNA was noticeable in older individuals (±70) [98]. In addition, HERV-K envelop protein was more highly expressed in African American patients (61%) than in European Americans (40%). Interestingly, the difference between the expression of HERV-K Gag mRNA in African American and European American prostatic patients was insignificant [98].

In fact, race, ethnicity, and geography could affect incidence of prostate cancer remarkably [152]. A recent metagenomic study revealed that Sardinian men have a lower risk of prostate cancer than other countries in Europe. Further analysis depicted that the incidence of ERG fusion, the most common genomic modification in prostate cancer patients [153], was less frequent among Sardinians. The lower rate of prostate cancer in this community might be related to over-expression of the UGT2B4 gene and consequent up-regulation of metabolism pathways, including ‘de novo’ IMP [152].

Analysis of the human genome revealed that HERV-K (HML1-10) transcripts existed in various cancers, such as prostate, breast, lung, melanoma, rheumatoid arthritis, and amyotrophic lateral sclerosis (ALS). Recently, a high proportion of HERV-K HML9 has been detected beyond the transcriptional segments dispersing between intergenic sites and introns [154]. In fact, integration of HERV proviruses inside the transcriptional sites condemned them to evolutional removal; therefore, they are embedded outside of transcriptional sites. Based on the evidence, HML-9 could influence various tissues according to the physiological circumstance and stage of malignancy through activating immune responses [154].

As a result of chromosomal translocation and amplification, abnormal up-regulation of ETS factors (i.e., *ERG*, *ETV1*, and *ETV4*) happens in solid tumors [155]. These oncogenic ETS factors have been detected in most prostate cancer resulting from the binding 5′untraslated segment of *TMPRSS2* to *ERG* [99]. Of newly discovered 5′partner, *TMPRSS2*, *SLC45A3*, *HERV-K_22q11.23*, *C15ORF21*, and *HNRPA2B1* need to be studied further. The analysis revealed that the ETS family contributes to relocating recurrent gene in prostate cancer [99].

LTRs contribute to carcinogenicity in many cancer types, in which they regulate expression of genes located next to them. HERV-K HML-2 has three subgroups: LTR5A, LTR5B, and LTR5H. Phylogenetic analysis disclosed the evolutional differences among these groups. The activity of the promotor was determined higher in LTR5H than in LTR5A and LTR5B. Analysis revealed that substitution of homologous fragments of LTR5B with homologous segments of an LTR5H that was divided into 4 pieces increased the activity of LTR5H. In addition, the transcriptional activity of LTR5H was enhanced by the presence of the TATA box and the two p53 binding sites in the LTR5H region. The combinational mutations in the TATA box and *P53* binding sites (*TP53*-1 and *TP53*-2) could remarkably reduce transcriptional profiles in LTR5H3-5B compared to the condition that only one of these sequences is involved in mutation [100]. To assess whether suppression of *TP53* via small interfering RNAs (siRNA1 and siRNA2) has any impact on the activity of LTR5H promotor, Western blot of p53 protein and qPCR analysis for estimating level of *P53* RNA expression in HEK293T/HeLa cells following luciferase reporter assays were carried out. The result depicted that siRNA2 had a higher intervening effect, in which muting *TP53* via siRNA markedly decreased the transcriptional activity of LTR5H promotor [100].

Eventually, this analysis depicted that p53 could influence the transcriptional profiles of HML-2 LTR5H. P53 is not only a tumor suppressor but it can also manage expression of genes associated with cell cycle arrest, organic processes, and apoptosis in response to cellular stress, and it is critical for control of homeostasis [100] (Table 1).

### 2.8. HERV-K and Atypical Teratoid Rhabdoid Tumors (AT/RT)

The result of a study demonstrated that deletion of the *SMARCB1* gene, a gene that inhibits tumors, triggers AT/RT (a central nervous system disease that causes hostile cells to be created in the brain [102]) and causes the *C-MYC* gene (a regulator/oncogene gene that contributes to expression of many genes, cell proliferation, and cancer [156]) to be translocated to HERV-K LTRs [103]. This resulted in activation of the Env gene in HERV-K HML-2 proviruses, production of ENV protein, and consequent liberation of this protein in extracellular vesicles. In normal conditions, attaching *SMARCB1* to a region next to the HML-2 promotor can lead to inhibition of HML-2 through DNA-protein precipitation. This suggests that the activators of the *SMARCB1* gene could probably be exploited in not only treatment of AT/RT but also inhibition of the HML-2 Env gene [103] (Table 1).

### 2.9. HERV-K and non-Hodgkin Lymphoma (NHL)

NHL is an abnormal mass generated in lymphoid tissue that is almost triggered by B cell precursors, mature B cells, T cell precursors, and mature T cells [104].

Expression of HERV-K DNA, RNA, and proteins has even been recognized in patients with NHL, suggesting a link between amount of HERV-K expression and severity or recurrence of NHL [105], in which the current FDA-approved medications against HIV that are normally administrated to NHLs patients reduced expression of HERV-K in these patients [105] (Table 1).

## 3. Therapeutic Strategies against Possible HERV-K-Induced Tumors

HERV-K immunogens could be exploited in various forms as possible treatments for HERV-K-induced tumors and cancers. In this regard, several studies have been carried out to evaluate the immunogenic responses of patients with tumors and cancers against HERV-K immunogens, such as Gag, Pol, Env, and reverse transcriptase inhibitors, etc. Some of the therapies against HERV-K-induced tumors and cancers are as follows:

### 3.1. HERV-K Gag-Based Vaccines

It has been proposed that different types of tumors might be well-responsive to vaccines against HERV-K Gag [157]. This is because HERV-K Gag could induce B cell responses in the human body through activation of autoantibodies. Experiments on murine renal carcinoma cells (Renca) that were transfected with HERV-K Gag (RLZ-HKGag) revealed that these genetically modified cells could produce Gag protein, and subdermal and intravenous administration of RLZ-HGag cells to syngeneic BALB/c mice led to local tumor and pulmonary metastases, respectively [157]. MVA-HK_con_ is an active vaccine against the HERV-K Gag protein. Surprisingly, the MVA-HK_con_ vaccine succeeded to reduce growth in tumors and number of nodules in pulmonary metastases that all were induced by RLZ-HKGag in experimental mice [157]. MVA-HK_con_ could highly induce T cell immune responses in vivo and knock out the cells positive to HERV-K taken from the same individuals. In terms of safety, MVA-HK_con_ is a low-risk vaccine that is made by the attenuated virus that is not able to replicate in the host cells, but it can express the protein in high-level concentration [157]. These data confirm that MVA-HK_con_ is efficient enough to suppress development of tumors. Accordingly, this vaccine could be administered as a tumor vaccine or substituted target for creating an HIV vaccine. Since HIV compromises the immune system, development of a low-risk vaccine made by HIV that is, in the meantime, effective against the virus could be challenging [157] (Table 2).

### 3.2. Antibody-Based Therapies

#### 3.2.1. Active Immunization against HERV-K as a Therapy against Solid Tumors

Human adenoviruses could be exploited in immunotherapy against solid tumors in order to carry HERV-K immunogens [158]. Earlier, a study depicted that an endogenous variant of murine leukemia that was transfected into adenoviral vectors could heal small-dimension cancers. In a recent investigation, engineered adenoviruses type 5 and 19a/64 that carried HERV-K Gag and Env genes underwent mutations in immunosuppressive domains (ISD) of Env glycoproteins [158]. Then, expression of HERV-K Gag and Env were evaluated in both human and murine DCs, followed by testing subsequent immunogenicity through intracellular cytokine staining and tetramers analyses [158]. At the next level, HERV-K-excited immune cells were collected from immunized mice and transferred to tumor-bearing mice (their murine colorectal cancer cells were genetically engineered to express the HERV-K Gag and Env antigens) [158]. Moreover, transfer of ISD-mutagenic HERV-K Gag and Env (HERV-K-ISDmut) via adenoviral carriers enhanced not only expression of HERV-K but also immune responses, including rapid antibody T cell responses in mice and immune tolerance breakdown in non-human primates [158]. Although failure of Gag- and Env-expressing cell lines happened in wild-type mice, transplanting CT26 cells excited by HERV-K Gag and Env to nude mice boosted the immune response in these animals. Of circulated CD8+ T cells, almost 40–50% are devoted to T cell responses. Further analysis suggested that a mix of CD8+ T cells and B cells (excited by HERV-K Gag and Env) impedes progress of tumors in the majority of the experimental animals promptly and continuously [158] (Table 2).

#### 3.2.2. Anti-HERV-K Env Protein Antibodies

Studies on breast cancer cell lines demonstrated that HERV-K Env protein normally appears on the surface of involved cells. Anti-HERV-K Env antibodies could be a promising immune therapy against tumor cancer [144]. To estimate whether anti-HERV-K Env antibodies would be effective against breast cancer, two breast cell panels, including cancerous ones (MDA-MB-231, MCF-7, SKBR3, MDA-MB-453, T47D, and ZR-75-1) and non-cancerous (MCF-10A and MCF-10AT), were selected and expression of HERV-K Env was assessed in these cell lines via immunoblot, ELISA, immunofluorescence [144]. In an in-vivo condition, in mice, the impact of anti-HERV-K Env monoclonal antibodies (mAbs; 6H5, 4D1, 4E11, 6E11, and 4E6) on expression of HERV-K Env in the targeted cell lines, dimension, and apoptosis of xenograft tumor cells were evaluated. The result of this research depicted that anti-HERV-K mAbs could restrain development of cancerous cells and prompt apoptosis of them in in vitro conditions. In in vivo conditions, in mice, 6H5 mAb diminished expansion of xenograft tumors rather than cancerous cell line of MDA-MB-231 exposed to 6H5 mAb and control immunoglobulin (control [mlgG]) [144]. In fact, mAbs could target T cell responses, and presence of cell surface molecules in human breast cancer could induce B cell responses.

Interestingly, the cytotoxic profile of 6H5 mAb impeded expansion of tumors in breast and ovarian cancer cells, including those formed in MCF-7 breast cancer and DOV13 ovarian cancer cell lines; however, no cytotoxicity was observed in benign breast cells and T80/T29 immortalized normal ovarian cells [159]. Further apoptosis analyses via Annexin V-APC and 7AAD-PEcy7 on a BD FACSArray bioanalyzer depicted that there is a direct association between titer of antibody and intensity of apoptosis in breast cancer cell lines except for benign breast cells [159]. Surprisingly, administration of 6H5 mAb attached to recombinant gelonin (rGel) turned it into a more cytotoxic compound (6H5/rGel) against MCF-7 cells (4.71-fold more than cells treated with only 6H5) [159].

Recently, an anti-HERV-K monoclonal antibody, GN_mAb_EnvK-01, has been invented that could attack a conserved and specific segment of HERV-K envelope, SLDKHKHKKLQSFYP, preventing it from glycosylation (it is essential for protein processing, receptor attachment, and immune escape [168]) and retaining its functional folded form (complex three-dimensional shape) [160]. These characteristics make this antibody an ideal agent for immunological diagnosis and treatment of HERV-K-associated diseases, such as ALS, etc. (Table 2).

#### 3.2.3. Anti-HERV-K Antibodies as The Future Breast Cancer Treatment?

A recent project has been trying to evaluate whether HERV-K would be able to influence the observable attributes in breast cancer through proliferation, cell migration and invasion, and colony formation analyses [169]. This ongoing project intends to employ commercial and natural anti-HERV-K antibodies along with protease inhibitors and putative HERV-K blockers to discover how vaccines composed of anti-HERV-K antibodies could defect immunosuppressive profiles of HERV-K in T cell, NK, and antigen presentation and T cell activation. The answers to this question could shed light on discovery of novel therapies effective against HERV-K-induced breast cancers [169].

### 3.3. HERV-K Inhibitors

#### 3.3.1. HERV-K Env Inhibitors

Copper is one of the important metal elements that exists in the structure of many enzymes (e.g., lysyl oxidase, cytochrome c oxidase, zinc–copper superoxide dismutase) as a cofactor [170,171]. Copper contributes to cell metabolism [170,171], supporting active robustness of epithelial and connective tissues/skin/blood vessels [172], generation of melatonin [173], and modulating cell signaling routes through its redox action [174]. In addition, copper has an equivocal role as antioxidant and prooxidant; it can cause free radicals or activate formation of ROS necessary for spread of cancers [175].

Recently, the impact of CuSo4 on transcription of various genes in three HERV families (H, K, and W) was assessed in patients with melanoma tumors. To find out the minimum concentrations of CuSo4 with lower cytotoxicity, melanoma SK-MEL-37 cells taken from malignant human skin were exposed to 25, 50, and 75 μM of CuSo4 for 96 hours [161]. Accordingly, the best concentration of CuSO4 that could remarkably decrease expression of HERV-H Env was estimated at 75 μM. This is under the condition that HERV-K Env and HERV-W Env were overexpressed at concentrations of 25 μM and all tested concentrations, respectively [161]. This result showed that an association existed between higher concentration of copper ions and regression of HERV-K Env or HERV-W proteins, leading to restriction in the tumor growth, whereas the lower concentration of copper enhanced production of Env protein in all three families and augmented the size of the tumor [161] (Table 2).

#### 3.3.2. HERV-K Reverse Transcriptase Inhibitors

The elevated level of endogenous reverse transcriptase (RT) has been linked to tumoral transformation of cells. The capability of non-nucleoside RT inhibitors (NNRTIs) in failing cell growth and cell diversification has been confirmed previously [162]. HERVs RT inhibitors, such as Abacavir (ABC: nucleoside RT inhibitors), could potentially be administered in treatment of cancers as they could impede the function of RT in HERVs [162]. ABC could decrease cell growth, spread, and invasiveness of tumoral cells in prostate cancer, which could lead to acceleration of aging and cell death [162]. In prostate cancer, ABC could postpone development of cell cycle S phase and influence growth of the tumor, in which cells started aging a couple of hours after application of ABC, and this was aligned with modification of cells morphologies and gradual rise in SA-β-gal expression in human prostate cell lines (PC3 and LNCaP) [162].

In PC3 cells, ABC could influence expression of specific genes that contributed to specific biological functions. This has been proven through IPA analysis on two concentrations of ABC. ABC-affected genes are involved in remodeling of chromatin through deceleration of S phase cell cycle and involving either endogenous or exogenous cells with several replicational strains [162].

On the other hand, ABC treatment could enhance the concentration of LINE-1 ORF1 and ORF2 transcripts in a dose–time-dependent manner. However, there are still ambiguities to prove that increase in quantity of ORF1 and ORF2 is related to expansion of LINE-2 cDNA or transcription of bona fida or stability of LINE-1 mRNA [162] (Table 2).

### 3.4. Clustered Regulatory Interspaced Short Palindromic Repeats (CRISPR)/Cas9 Technology

Clustered regulatory interspaced short palindromic repeats (CRISPR)/Cas9 technology could also be recruited in treatment of cancers and tumors. This technology was tried out on prostate cell line LNCaP using the Cas9 system for *Staphylococcus aureus* (SaCas9) and resulted in impairment of HERV-K (HML-2) Env gene, inhibiting its transcription and translation [176]. Further analysis depicted that damage of HERV-K (HML-2) Env gene impedes function of several fundamental regulators that contribute to RNA binding and splicing, including epidermal growth factor receptor (EGF-R), nuclear factor kappa-light-chain-enhancer of activated B cells (NF-kB), splicing factor 2/alternative splicing factor (SF2/ASF), and TAR-DNA-binding-protein-43 (TDP-43) [176]. This result underlined the potential contribution of HERV-K in cancers/tumors, shedding light on development of novel approaches for treatments of cancers [176].

CRISPR-Cas9 knock-out (KO) of the HERV-K Env gene was also exploited to assess expression of HERV-K Env protein and spread and aggressiveness of ovarian cancer in SKOV3 and OVCAR3 cell lines [163]. The result of a study showed that the HERV-K Env gene KO strategy could influence growth, invasiveness, and general dissemination of ovarian tumors via regulation of RB and Cyclin B1 proteins based on the cell types. On the one hand, HERV-K Env gene KO diminished production of HERV-K Env RNA and its protein and decreased spread and aggression of affected cells and tumors [163]. Furthermore, up-regulation of RB protein and down-regulation of cyclin B1 proteins were prominent in HERV-K Env KO SKOV3. Contrary down-regulation of phosphor-RB was also reported in HERV-K Env KO OVCAR3 cells [163].

In AT/RT cases, application of CRISPR-dead CAS9 (dCAS9: a mutant form of CAS without endonuclease and commonly used in CRISPR-Cas technology [177]) along with suppressor proteins could down-regulate expression of HERV-K HML-2 and decrease cell multiplication, leading to apoptosis in vitro. This is under the condition that application of CRISPR-dCAS9 along with siRNA and short hairpin RNA (shRNA) could lead to not only HERV-K HML-2 knock-out but also extreme regression of Ras protein [103]. In a recent study, a CRISPi-based structure was engineered containing a lentiviral vector with CRISPR-dCas9 and four fused Sin3 repressive interacting domains (SID) with or without four gRNAs targeting HML-2 LTR5_Hs (SID4X). The engineered CRISPi with or without gRNA could influence HERV-K LTR5_Hs and decrease transcription of HERV-K Env significantly [103] (Table 2).

### 3.5. Anti-HERV-K Expression

#### 3.5.1. Anti-HIV-1 Drugs

Some HIV-1 reverse transcriptase inhibitors, such as lamivudine (3TC), zidovudine (AZT), and tenofovir disoproxil fumarate (TDF), could generally restrain retrotransposons and HERVs, preventing HERV-K expression [164]. However, some other anti-HIV drugs, such as Atazanavir, an HIV protease inhibitor, could decrease expression of HERV-K [158].

The result of ongoing research on the efficiency of anti-HIV-retroviral medications, effective for ALS, including darunavir, ritonavir, dolutegravir, and tenofovir alafenamide (TAF), on HERV-K suppression revealed that these drugs diminished load of HERV-K after 24 weeks through quantitative-PCR analysis [178].

The current FDA-approved medications against HIV that are normally administrated to NHLs patients could also reduce expression of HERV-K in these patients [105]. In one of the recent studies, both lamivudine and tenofovir disoproxil fumarate (tenofovir), the current FDA-approved medications against HIV, were administrated to NHLs patients aiming to understand whether these HERV-K inhibitory drugs would be effective against NHLs or could prohibit recurrence of the disease to assess the impact of lamivudine and tenofovir in combination on the concentration of HERV-K RNA released in plasma and to discover the side effects and safety of taking these drugs together in NHLs patients [105]. This investigation revealed that lamivudine and tenofovir in combination could decrease expression of HERV-K in NHLs patients [105]. Further studies are needed to confirm whether suppression of HERV-K could impact control and recurrence of NHLs (Table 2).

#### 3.5.2. Anti-Coronavirus Drugs

Excessive expression of HERV-K was observed in respiratory tracts of SARS-CoV-2 cases in addition to plasma of patients who died due to SARS-CoV-2 rather than individuals shedding the virus and healthy controls [164]. An analysis of patients who died due to SARS-CoV-2 depicted that some factors enhanced expression of HERV-K in these patients, such as overexpression of proinflammatory markers, induction of monocytes, and escalated use of clotting factors (they can increase risk of hemorrhage [179]). In fact, expression of HERV-K was induced by SARS-CoV-2 in human primary monocytes. Experiments on the plasma taken from dead patients revealed that expression of HERV-K lessened levels of regulatory or anti-inflammatory responses, such as IL-1Ra and IL-13, in these cases, which could, consequently, impede activation of IL-1 and excite allergic-like or TH2 signals [180,181]. In contrast, HERV-K induced generation of some proinflammatory responses, such as IL-17 and probably IL-6, CRP [182] in dead patients with autoimmune diseases [183]. Patients with higher expression of HERV-K LTR protein experienced declined level of IL-13 generation and repression in CD4 and CD8 T cell responses [182]. In fact, SARS-CoV-2 could replicate its genome in the infected monocytes, but the infection may not result in production of infectious particles. Pyroptosis of the involved monocytes led to production of pro-inflammatory responses along with a cytokine storm [184,185,186,187,188]. Anti-coronavirus drug remdesivir (RDV), a nucleotide prodrug of an adenosine C-nucleotide analog [165], could halt replication of SARS-CoV-2 RNA in monocytes [166] in addition to expression of HERV-K that was induced by SARS-CoV-2. While steroidal anti-inflammatory drugs such as dexamethasone and prednisolone boosted HERV-K depletion, recombinant anti-TNF drugs fused to the constant end (FC) of immunoglobulin impeded HERV-K expression to some extent [164] (Table 2).

## 4. Conclusions

The contribution of HERV-K subclass in tumorigenicity/carcinogenicity has been suggested by a variety of evidence, including the presence of intact ORFs in HERV-K for expression of essential retroviral immunogens, such as Gag, Pol, and Env, in which impairment of HERV-K ORFs and tumor suppressor genes could influence expression of these immunogens; capability of HERV-K in conversion of silent proto-oncogenes to cancerous cells; overexpression of HERV-K immunogens in tumor-involved areas, which leads to production of retroviral RNAs, functional proteins, and retroviral-like particles; the association between overexpression of HERV-K in specific cell lines (i.e., embryonal carcinoma, teratoma) considering age, race, and smoking status of patients with tumors (i.e., prostate cancer); hypomethylation of HERV-K, specifically in clinical and chronic stages of malignancy; the impact of HERV-K on emerging new promotors and altering transcription patterns following generation of new mRNAs; conversion of HERV-K polyproteins into structural or enzymatic subunits under viral or host protease exposure that ends in liberation of HERV-K particles from tumor cell lines (such as teratoma cell lines, i.e., TM and RM) and suppression of immune responses through modifying several cytokines and cellular genes; direction of higher titers of antibodies, such as IgG against HERV-K particles (i.e., Gag/Env/Rec/N9) in ovarian, testicular (GCT), prostate, breast cancers, and lymphoma tumors; and the direct influence of HERV-K Env on modulation of the stress-signaling NUPR-Rb pathway. Surprisingly, wherever cancerous and tumor cells existed (they principally present in germ cells, skin, teratoma, lymph nodes, and breast, ovary, and prostate), HERV-K is able to express better and induce higher immune responses (antibody titer) in involved patients than normal individuals. However, concluding that HERV-K and its products are the initial tumor inducers or simply play roles in tumor dispersal is an intricate debate.

The most common vaccines and therapies have immunomodulatory impacts on HERV-K-associated tumors, in which they either induce T cell responses and apoptosis or suppress HERV-K expression, aggressive autoimmune responses, and tumor growth.

Eventually, to discover more rapid and effective therapies against HERV-K-associated tumors and cancers, more research is needed to explore the precise role of HERV-K immunogens in onset and progress of various malignancies. This should be followed by careful examination of all novel and common therapeutic options, individually or within synergistic therapies, against all types of tumors.

## Figures and Tables

**Table 1:** Possible tumorigenicity of HERV-K particles in various organs or tissues, along with tumor risk factors and evidence of HERV-K particles in tumors.

Tumor Type	Involved Organs or Tissues	Tumor Risk Factors	Evidence of HERV-K Particles
**Melanoma**	Skin [70]	Expression/methylation of LINE-1, methylation of HERV-K [75]	HERV-K Gag, Pol, Env, and Rec proteins [76]
**Teratocarcinoma**	mixed germ-line tumors: female sexual gonads, placenta, extraembryonic membranes, and umbilical cord [82,83]	Changing the expression pattern of cytokines and cellular genes due to teratoma-liberated viral proteins [46], deglycosylation of HML-2 GH and Tera-1 [84]	TM ^1^, HERV-K Env [46]
**Osteosarcoma**	Bones	Dysfunctionality of TE ^2^ and DNA damages, overexpression of LINE-1/Alu/SVA/HERV-K [85]	Not yet defined
**Colorectal cancer (CRC)**	Colon and rectum	Conversion of NUPR1 into a chromatin protein due to stress condition [86], lower methylation rate of LINE-1 or HERV, EVs ^3^ [87]	HERV-K Env transcripts class II [88]
**Breast tumors**	Breast [89]	Activation of Ras-dependent ERK ^4^ 1/2 mitogen-activated protein (MAP) kinase pathway and its transformation [90]	HML-2 Env mRNA [38,91], transcripts of Gag and Env genes [91]
**Ovarian cancer**	Ovary [92]	Induction of immunity by HERV-K Env protein and production of T cells, INFγ, and HERV-K-specific CTL ^5^ [93].	HERV-K transcripts, Env protein, and active reverse transcriptase [93]
**Prostate cancer**	Prostate [94]	Expression of the *NGO-Pr-54* region (a Gag-related antigen) [95], encoding chromosome 22q11.23 by HERV-K Gag proteins [96], overexpression of HERV-K_22q11.23 and accessory Np9 [97], elevated level of interferon-γ and expression of HERV-K Gag [98], chromosomal translocation and amplification, abnormal up-regulation of ETS factors [99], transcriptional activity of HERV-K LTRs [100]	HERV-K Gag and Gag RNA [101], HERV-K Env [98],
**Atypical teratoid rhabdoid tumors (AT/RT)**	Central nervous system (brain) [102]	Deletion of the *SMARCB1* gene [103]	HERV-K Env [103]
**Non-Hodgkin lymphoma (NHL)**	Lymphoid tissue [104]	HERV-K expression and its impact in severity or recurrence of NHL [105]	HERV-K DNA, RNA, and proteins [105]

^1^ TM: A type of recombinant HERV-K transmembrane envelope. ^2^ TE: Transposable elements. ^3^ EVs: Extracellular vesicles. ^4^ ERK: Extracellular-signal-regulated kinase. ^5^ CTL: Cytotoxic T lymphocytes.

**Table 2:** The roles of various therapies in expression of HERV-K particles in tumors and consequent immune responses.

Types of Therapy	HERV-K Target	Immune Response	Impact on Tumor
MVA-HK_con_ vaccine [157]	Gag [157]	Inducing the T cell immune responses, knocking out the cells positive to HERV-K [157]	Suppressing growth of the tumor and reducing pulmonary metastases [157]
Active immunization via engineered adenovirus type 5 and 19a/64 carrying ISD-mutant HERV-K Gag and Env [158]	Gag and Env [158]	Rapid antibody T cell responses in mice, immune tolerance breakdown in non-human primates [158]	Suppressing growth of tumors [158]
Anti-HERV-K Env monoclonal antibodies [144]	Env [144]	mAbs ^1^ Env and T cell immune response in breast cancer, high number of cell surface molecules in human breast cancer that target the B cell response [144]	mAbs: generally restrained development of cancerous cells and promoted apoptosis of them in in vitro conditions [144]. 6H5 mAb: diminished expansion of xenograft tumors due to its cytotoxic profile [159]. GN_mAb_EnvK-01: treatment of HERV-K-associated diseases, such as ALS [160]
HERV-K Env inhibitors, such as CuSo4 [161]	Env [161]	Not yet defined	Higher concentration of CuSo4 and restriction of tumor growth [161]
HERV-K reverse transcriptase inhibitors, such as ABC ^2^ [162]	RT ^3^ [162]	Accelerating aging and cell death, affecting the genes responsible for remodeling of chromatin [162]	Declining cell growth/spread/ invasiveness of tumoral cells in prostate cancer [162]
CRISPR ^4^/Cas9 technology	Env	HERV-K HML-2 knock-out, regression of Ras protein [103], regulating RB and Cyclin B1 proteins, declining the cell multiplication, and inducing apoptosis in vitro [163]	Suppressing growth, invasiveness, and dissemination of ovarian tumors [163]
Anti-HIV-1 RT drugs [105]	Expression of HERV-K in general [105]	Not yet defined	Preventing HERV-K expression (such as lamivudine, zidovudine, etc.), Inhibiting HERV-K expression (Atazanavir) [105]
Anti-coronavirus drugs (A) RDV [164]	Replication of SARS-CoV-2 RNA [165] and expression of HERV-K	Inhibiting inflammatory responses [166]	Not defined yet
(B) Steroidal anti-inflammatory drugs (e.g., dexamethasone, prednisolone) [164]	HERV-K expression [164]	Inhibiting inflammatory responses [164]	Not defined yet
(C) Anti-TNF drugs fused to the constant end (FC) of immunoglobulin [164]	HERV-K expression [164]	Targeting TNF and ceasing the inflammatory responses [167]	Not defined yet

^1^ mAb: Monoclonal antibodies. ^2^ ABC: Abacavir. ^3^ RT: Reverse transcriptase. ^4^ CRISPR: Clustered regulatory interspaced short palindromic repeats.

## Data Availability

No new data was generated; all material is available upon request.

## References

[1] Venkatesan A., Johnson R.T., Goodin D.S. (2014). Infections and multiple sclerosis. Multiple Sclerosis and Related Disorders.

[2] Luzuriaga K., Long S.S. (2012). Introduction to retroviridae. Principles and Practice of Pediatric Infectious Diseases.

[3] Grandi N., Tramontano E. (2018). Human endogenous retroviruses are ancient acquired elements still shaping innate immune responses. Front. Immunol..

[4] Nelson P.N., Carnegie P.R., Martin J., Davari Ejtehadi H., Hooley P., Roden D., Rowland-Jones S., Warren P., Astley J., Murray P.G. (2003). Demystified. Human endogenous retroviruses. Mol. Pathol..

[5] Bannert N., Kurth R. (2006). The evolutionary dynamics of human endogenous retroviral families. Annu. Rev. Genomics Hum. Genet..

[6] Pisano M.P., Grandi N., Cadeddu M., Blomberg J., Tramontano E. (2019). Comprehensive characterization of the human endogenous retrovirus HERV-K(HML-6) Group: Overview of structure, phylogeny, and contribution to the human genome. J. Virol..

[7] Alcazer V., Bonaventura P., Depil S. (2020). Human endogenous retroviruses (HERVs): Shaping the innate immune response in cancers. Cancers.

[8] Smit A.F.A. (1999). Interspersed repeats and other mementos of transposable elements in mammalian genomes. Curr. Opin. Genet. Dev..

[9] (1998). Larsson; Andersson beneficial role of human endogenous retroviruses: Facts and hypotheses. Scand. J. Immunol..

[10] Nelson P.N., Hooley P., Roden D., Davari Ejtehadi H., Rylance P., Warren P., Martin J., Murray P.G. (2004). Human endogenous retroviruses: Transposable elements with potential?. Clin. Exp. Immunol..

[11] Blomberg J., Ushameckis D., Jern P. (2008). Evolutionary aspects of human endogenous retroviral sequences (HERVs) and disease. Madame Curie Bioscience Database.

[12] Gröger V., Cynis H. (2018). Human endogenous retroviruses and their putative role in the development of autoimmune disorders such as multiple sclerosis. Front. Microbiol..

[13] Boller K., König H., Sauter M., Mueller-Lantzsch N., Löwer R., Löwer J., Kurth R. (1993). Evidence that HERV-K is the endogenous retrovirus sequence that codes for the human teratocarcinoma-derived retrovirus HTDV. Virology.

[14] Löwer R., Boller K., Hasenmaier B., Korbmacher C., Müller-Lantzsch N., Löwer J., Kurth R. (1993). Identification of human endogenous retroviruses with complex MRNA expression and particle formation. Proc. Natl. Acad. Sci. USA.

[15] Nelson P.N. (1995). Retroviruses in rheumatic diseases. Ann. Rheum. Dis..

[16] Nelson P.N., Lever A.M.L., Smith S., Pitman R., Murray P., Perera S.A., Westwood O.M.R., Hay F.C., Ejtehadi H.D., Booth J.C. (1999). Molecular investigations implicate human endogenous retroviruses as mediators of anti-retroviral antibodies in autoimmune rheumatic disease. Immunol. Investig..

[17] Wilkinson D., Mager D., Leong J., Levy J.A. (1994). Endogenous human retroviruses. The Retroviridae.

[18] Subramanian R.P., Wildschutte J.H., Russo C., Coffin J.M. (2011). Identification, characterization, and comparative genomic distribution of the HERV-K (HML-2) group of human endogenous retroviruses. Retrovirology.

[19] Perl A., Rosenblatt J.D., Chen I.S.Y., DiVincenzo J.P., Bever R., Poiesz J., Abraham G.N. (1989). Detection and cloning of new HTLV-related endogenous sequences in man. Nucleic Acids Res..

[20] Löwer R., Löwer J., Kurth R. (1996). The viruses in all of us: Characteristics and biological significance of human endogenous retrovirus sequences. Proc. Natl. Acad. Sci. USA.

[21] Downey R.F., Sullivan F.J., Wang-Johanning F., Ambs S., Giles F.J., Glynn S.A. (2015). Human endogenous retrovirus K and cancer: Innocent bystander or tumorigenic accomplice?. Int. J. Cancer.

[22] Chan S.M., Sapir T., Park S.-S., Rual J.-F., Contreras-Galindo R., Reiner O., Markovitz D.M. (2019). The HERV-K accessory protein Np9 controls viability and migration of teratocarcinoma cells. PLoS ONE.

[23] Ellinghaus D., Kurtz S., Willhoeft U. (2008). LTRharvest, an efficient and flexible software for de novo detection of LTR retrotransposons. BMC Bioinform..

[24] Guffanti G., Bartlett A., DeCrescenzo P., Macciardi F., Hunter R., Binder E.B., Klengel T. (2019). Transposable elements. Behavioral Neurogenomics.

[25] Sarah P., Guy O., François M. (2004). A retroviral promoter and a cellular enhancer define a bipartite element which controls Env ERVWE1 placental expression. J. Virol..

[26] Seifarth W., Frank O., Zeilfelder U., Spiess B., Greenwood A.D., Hehlmann R., Leib-Mösch C. (2005). Comprehensive analysis of human endogenous retrovirus transcriptional activity in human tissues with a retrovirus-specific microarray. J. Virol..

[27] van de Lagemaat L.N., Landry J.-R., Mager D.L., Medstrand P. (2003). Transposable elements in mammals promote regulatory variation and diversification of genes with specialized functions. Trends Genet..

[28] Wang J., Xie G., Singh M., Ghanbarian A.T., Raskó T., Szvetnik A., Cai H., Besser D., Prigione A., Fuchs N.V. (2014). Primate-specific endogenous retrovirus-driven transcription defines naive-like stem cells. Nature.

[29] Andersson A.C., Svensson A.C., Rolny C., Andersson G., Larsson E. (1998). Expression of human endogenous retrovirus ERV3 (HERV-R) mRNA in normal and neoplastic tissues. Int. J. Oncol..

[30] Sauter M., Schommer S., Kremmer E., Remberger K., Dölken G., Lemm I., Buck M., Best B., Neumann-Haefelin D., Mueller-Lantzsch N. (1995). Human endogenous retrovirus K10: Expression of Gag protein and detection of antibodies in patients with seminomas. J. Virol..

[31] Löwer R., Löwer J., Tondera-Koch C., Kurth R. (1993). A general method for the identification of transcribed retrovirus sequences (R-U5 PCR) reveals the expression of the human endogenous retrovirus loci HERV-H and HERV-K in teratocarcinoma cells. Virology.

[32] Schulte A.M., Lai S., Kurtz A., Czubayko F., Riegel A.T., Wellstein A. (1996). Human trophoblast and choriocarcinoma expression of the growth factor pleiotrophin attributable to germ-line insertion of an endogenous retrovirus. Proc. Natl. Acad. Sci. USA.

[33] Bera T.K., Tsukamoto T., Panda D.K., Huang T., Guzman R.C., Hwang S.-I., Nandi S. (1998). Defective retrovirus insertion activates C-Ha-Ras proto-oncogene in an MNU-induced rat mammary carcinoma. Biochem. Biophys. Res. Commun..

[34] Ahn K., Kim H.S. (2009). Structural and quantitative expression analyses of HERV gene family in human tissues. Mol. Cells.

[35] Yang C., Guo X., Li J., Han J., Jia L., Wen H.L., Sun C., Wang X., Zhang B., Li J. (2022). Significant upregulation of HERV-K (HML-2) transcription levels in human lung cancer and cancer cells. Front. Microbiol..

[36] Tönjes R.R., Löwer R., Boller K., Denner J., Hasenmaier B., Kirsch H., König H., Korbmacher C., Limbach C., Lugert R. (1996). HERV-K: The biologically most active human endogenous retrovirus family. JAIDS J. Acquir. Immune Defic. Syndr..

[37] Barbulescu M., Turner G., Seaman M.I., Deinard A.S., Kidd K.K., Lenz J. (1999). Many human endogenous retrovirus K (HERV-K) proviruses are unique to humans. Curr. Biol..

[38] Etkind P.R., Lumb K., Du J., Racevskis J. (1997). Type 1 HERV-K genome is spliced into subgenomic transcripts in the human breast tumor cell line T47D. Virology.

[39] Srinivasachar Badarinarayan S., Sauter D. (2022). Not all viruses cause disease: HERV-K(HML-2) in healthy human tissues. PLoS Biol..

[40] Dervan E., Bhattacharyya D.D., McAuliffe J.D., Khan F.H., Glynn S.A. (2021). Ancient adversary—HERV-K (HML-2) in cancer. Front. Oncol..

[41] Dupressoir A., Lavialle C., Heidmann T. (2012). From ancestral infectious retroviruses to bona fide cellular genes: Role of the captured syncytins in placentation. Placenta.

[42] Jean-Luc B., Dimitri L., Valérie C., Olivier B., Guy O., Sylvie C.-F., Bernard M., François M., François-Loïc C. (2000). An envelope glycoprotein of the human endogenous retrovirus HERV-W is expressed in the human placenta and fuses cells expressing the type D mammalian retrovirus receptor. J. Virol..

[43] Mi S., Lee X., Li X., Veldman G.M., Finnerty H., Racie L., LaVallie E., Tang X.-Y., Edouard P., Howes S. (2000). Syncytin is a captive retroviral envelope protein involved in human placental morphogenesis. Nature.

[44] Blaise S., de Parseval N., Bénit L., Heidmann T. (2003). Genomewide screening for fusogenic human endogenous retrovirus envelopes identifies syncytin 2, a gene conserved on primate evolution. Proc. Natl. Acad. Sci. USA.

[45] Best S., Le Tissier P., Towers G., Stoye J.P. (1996). Positional cloning of the mouse retrovirus restriction gene Fvl. Nature.

[46] Morozov V.A., Dao Thi V.L., Denner J. (2013). The transmembrane protein of the human endogenous retrovirus—K (HERV-K) modulates cytokine release and gene expression. PLoS ONE.

[47] Bieda K., Hoffmann A., Boller K. (2001). Phenotypic heterogeneity of human endogenous retrovirus particles produced by teratocarcinoma cell lines. J. Gen. Virol..

[48] Gotzinger N., Sauter M., Roemer K., Mueller-Lantzsch N. (1996). Regulation of human endogenous retrovirus-K Gag expression in teratocarcinoma cell lines and human tumours. J. Gen. Virol..

[49] Depil S., Roche C., Dussart P., Prin L. (2002). Expression of a human endogenous retrovirus, HERV-K, in the blood cells of leukemia patients. Leukemia.

[50] Brodsky I., Foley B., Gillespie D. (1993). Expression of human endogenous retrovirus (HERV-K) in chronic myeloid leukemia. Leuk. Lymphoma.

[51] Brodsky I., Foley B., Haines D., Johnston J., Cuddy K., Gillespie D. (1993). Expression of HERV-K proviruses in human leukocytes. Blood.

[52] Cowan A.J., Green D.J., Kwok M., Lee S., Coffey D.G., Holmberg L.A., Tuazon S., Gopal A.K., Libby E.N. (2022). Diagnosis and management of multiple myeloma: A review. JAMA.

[53] Oganesyan A., Gregory A., Malard F., Ghahramanyan N., Mohty M., Kazandjian D., Mekinian A., Hakobyan Y. (2022). Monoclonal gammopathies of clinical significance (MGCS): In pursuit of optimal treatment. Front. Immunol..

[54] Masuda Y., Ishihara R., Murakami Y., Watanabe S., Asao Y., Gotoh N., Kasamatsu T., Takei H., Kobayashi N., Saitoh T. (2022). Clinical significance of human endogenous retrovirus K (HERV-K) in multiple myeloma progression. Int. J. Hematol..

[55] Kleiman A., Senyuta N., Trjakin A., Vinogradova T., Karseladze A., Gurtsrvitch V., Tjulandin S., Harnden P., Joffe J.K., Jones W.G. (2002). Expression of human endogenous retroviruses HERV-K/HTDV in germ cell tumours: Possible biological role and clinical application. Germ Cell Tumours V..

[56] Xue B., Zeng T., Jia L., Yang D., Lin S.L., Sechi L.A., Kelvin D.J. (2020). Identification of the distribution of human endogenous retroviruses K (HML-2) by PCR-based target enrichment sequencing. Retrovirology.

[57] Lu X., Sachs F., Ramsay L., Jacques P.-É., Göke J., Bourque G., Ng H.-H. (2014). The retrovirus HERVH Is a long noncoding RNA required for human embryonic stem cell identity. Nat. Struct. Mol. Biol..

[58] Durruthy-Durruthy J., Sebastiano V., Wossidlo M., Cepeda D., Cui J., Grow E.J., Davila J., Mall M., Wong W.H., Wysocka J. (2016). The primate-specific noncoding RNA HPAT5 regulates pluripotency during human preimplantation development and nuclear reprogramming. Nat. Genet..

[59] Fuentes D.R., Swigut T., Wysocka J. (2018). Systematic perturbation of retroviral LTRs reveals widespread long-range effects on human gene regulation. eLife.

[60] Zhang Y., Li T., Preissl S., Amaral M.L., Grinstein J.D., Farah E.N., Destici E., Qiu Y., Hu R., Lee A.Y. (2019). Transcriptionally active HERV-H retrotransposons demarcate topologically associating domains in human pluripotent stem cells. Nat. Genet..

[61] Jansz N., Faulkner G.J. (2021). Endogenous retroviruses in the origins and treatment of cancer. Genome Biol..

[62] Kaufmann S., Sauter M., Schmitt M., Baumert B., Best B., Boese A., Roemer K., Mueller-Lantzsch N. (2010). Human endogenous retrovirus protein Rec interacts with the testicular Zinc-finger protein and androgen receptor. J. Gen. Virol..

[63] Grow E.J., Flynn R.A., Chavez S.L., Bayless N.L., Wossidlo M., Wesche D.J., Martin L., Ware C.B., Blish C.A., Chang H.Y. (2015). Intrinsic retroviral reactivation in human preimplantation embryos and pluripotent cells. Nature.

[64] Kassiotis G. (2014). Endogenous retroviruses and the development of cancer. J. Immunol..

[65] Hanke K., Hohn O., Bannert N. (2016). HERV-K(HML-2), a seemingly silent subtenant—but still waters run deep. APMIS.

[66] Turner G., Barbulescu M., Su M., Jensen-Seaman M.I., Kidd K.K., Lenz J. (2001). Insertional polymorphisms of full-length endogenous retroviruses in humans. Curr. Biol..

[67] Schulz W.A. (2017). Does HERV-K represent a potential therapeutic target for prostate cancer?. Expert. Opin. Ther. Targets.

[68] Wright C.J., Smith C.W.J., Jiggins C.D. (2022). Alternative splicing as a source of phenotypic diversity. Nat. Rev. Genet..

[69] Nooraei S., Bahrulolum H., Hoseini Z.S., Katalani C., Hajizade A., Easton A.J., Ahmadian G. (2021). Virus-like particles: Preparation, immunogenicity and their roles as nanovaccines and drug nanocarriers. J. Nanobiotechnology.

[70] Davis L.E., Shalin S.C., Tackett A.J. (2019). Current state of melanoma diagnosis and treatment. Cancer Biol. Ther..

[71] Hahn S., Ugurel S., Hanschmann K.M., Strobel H., Tondera C., Schadendorf D., Löwer J., Löwer R. (2008). Serological response to human endogenous retrovirus K in melanoma patients correlates with survival probability. AIDS Res. Hum. Retrovir..

[72] Schiavetti F., Thonnard J., Colau D., Boon T., Coulie P. (2002). A human endogenous retroviral sequence encoding an antigen recognized on melanoma by cytolytic T lymphocytes. Cancer Res..

[73] Qi X., Sandmeyer S., Zaher H., Jez J. (2021). DNA repair | nonhomologous recombination: Retrotransposons. Encyclopedia of Biological Chemistry III.

[74] Briggs E.M., Ha S., Mita P., Brittingham G., Sciamanna I., Spadafora C., Logan S.K. (2018). Long interspersed nuclear element-1 expression and retrotransposition in prostate cancer cells. Mob. DNA.

[75] Cardelli M., van Doorn R., Larcher L., Donato M.D., Piacenza F., Pierpaoli E., Giacconi R., Malavolta M., Rachakonda S., Gruis N.A. (2020). Association of HERV-K and LINE-1 hypomethylation with reduced disease-free survival in melanoma patients. Epigenomics.

[76] Muster T., Waltenberger A., Grassauer A., Hirschl S., Caucig P., Romirer I., Födinger D., Seppele H., Schanab O., Magin-Lachmann C. (2003). An endogenous retrovirus derived from human melanoma cells. Cancer Res..

[77] Büscher K., Trefzer U., Hofmann M., Sterry W., Kurth R., Denner J. (2005). Expression of human endogenous retrovirus K in melanomas and melanoma cell lines. Cancer Res..

[78] Schmitt K., Reichrath J., Roesch A., Meese E., Mayer J. (2013). Transcriptional profiling of human endogenous retrovirus group HERV-K(HML-2) loci in melanoma. Genome Biol. Evol..

[79] Singh M., Cai H., Bunse M., Feschotte C., Izsvák Z. (2020). Human endogenous retrovirus K Rec forms a regulatory loop with MITF that opposes the progression of melanoma to an invasive stage. Viruses.

[80] Miriam D., Marlies S., Vivienne A., Licht J.D., Klaus R., Nikolaus M.-L. (2007). Physical and functional interactions of human endogenous retrovirus proteins Np9 and Rec with the promyelocytic leukemia zinc finger protein. J. Virol..

[81] Kirsten H., Oliver H., Linda L., Katharina F., Jula W., Reinhard K., Norbert B. (2013). Staufen-1 interacts with the human endogenous retrovirus family HERV-K(HML-2) Rec and Gag proteins and increases virion production. J. Virol..

[82] Damjanov I. (1990). Teratocarcinoma stem cells. Cancer Surv..

[83] Thowfeequ S., Srinivas S. (2022). Embryonic and extraembryonic tissues during mammalian development: Shifting boundaries in time and space. Philos. Trans. R. Soc. B Biol. Sci..

[84] Morozov V.A., Morozov A.V. (2021). A comprehensive analysis of human endogenous retroviruses HERV-K (HML.2) from teratocarcinoma cell lines and detection of viral cargo in microvesicles. Int. J. Mol. Sci..

[85] Wang C., Liang C. (2022). The insertion and dysregulation of transposable elements in osteosarcoma and their association with patient event-free survival. Sci. Rep..

[86] Garcia-Montero A., Vasseur S., Mallo G.V., Soubeyran P., Dagorn J.C., Iovanna J.L. (2001). Expression of the stress-induced P8 MRNA is transiently activated after culture medium change. Eur. J. Cell Biol..

[87] Ferrari L., Cafora M., Rota F., Hoxha M., Iodice S., Tarantini L., Dolci M., Delbue S., Pistocchi A., Bollati V. (2019). Extracellular vesicles released by colorectal cancer cell lines modulate innate immune response in zebrafish model: The possible role of human endogenous retroviruses. Int. J. Mol. Sci..

[88] Willer A., Saußele S., Gimbel W., Zeifarth W., Kister P., Leib-Mo¨sch C., Hehlmann R. (1997). Two groups of endogenous MMTV related retroviral Env transcripts expressed in human tissues. Virus Genes.

[89] Alkabban F., Ferguson T. (2022). Breast cancer. StatPearls.

[90] Lemaître C., Tsang J., Bireau C., Heidmann T., Dewannieux M. (2017). A Human endogenous retrovirus-derived gene that can contribute to oncogenesis by activating the ERK pathway and inducing migration and invasion. PLoS Pathog..

[91] Contreras-Galindo R., Kaplan M., Leissner P., Verjat T., Ferlenghi I., Bagnoli F., Giusti F., Dosik M., Hayes D., Gitlin S. (2008). Human endogenous retrovirus K (HML-2) elements in the plasma of people with lymphoma and breast cancer. J. Virol..

[92] Roett MA E.P. (2009). Ovarian Cancer: An Overview. Am. Fam. Physician.

[93] Rycaj K., Plummer J.B., Yin B., Li M., Garza J., Radvanyi L., Ramondetta L.M., Lin K., Johanning G.L., Tang D.G. (2015). Cytotoxicity of human endogenous retrovirus K–specific T cells toward autologous ovarian cancer cells. Clin. Cancer Res..

[94] Stephen W.L., Taylor L., Soon-Sutton Anu R.I., Sajjad H., Siref L.E. (2022). Prostate cancer. StatPearls.

[95] Ishida T., Obata Y., Ohara N., Matsushita H., Sato S., Uenaka A., Saika T., Miyamura T., Chayama K., Nakamura Y. (2008). Identification of the HERV-K Gag antigen in prostate cancer by SEREX using autologous patient serum and its immunogenicity. Cancer Immun..

[96] Johanning G.L., Malouf G.G., Zheng X., Esteva F.J., Weinstein J.N., Wang-Johanning F., Su X. (2017). Expression of human endogenous retrovirus-K is strongly associated with the basal-like breast cancer phenotype. Sci. Rep..

[97] Goering W., Ribarska T., Schulz W.A. (2011). Selective changes of retroelement expression in human prostate cancer. Carcinogenesis.

[98] Wallace T.A., Downey R.F., Seufert C.J., Schetter A., Dorsey T.H., Johnson C.A., Goldman R., Loffredo C.A., Yan P., Sullivan F.J. (2014). Elevated HERV-K mRNA expression in PBMC is associated with a prostate cancer diagnosis particularly in older men and smokers. Carcinogenesis.

[99] Helgeson B.E., Tomlins S.A., Shah N., Laxman B., Cao Q., Prensner J.R., Cao X., Singla N., Montie J.E., Varambally S. (2008). Characterization of TMPRSS2:ETV5 and SLC45A3:ETV5 gene fusions in prostate cancer. Cancer Res..

[100] Mengying L., Lei J., Hanping L., Yongjian L., Jingwan H., Xiaolin W., Tianyi L., Jingyun L., Bohan Z., Xiuli Z. (2022). P53 binding sites in long terminal repeat 5Hs (LTR5Hs) of human endogenous retrovirus K family (HML-2 subgroup) play important roles in the regulation of LTR5Hs transcriptional activity. Microbiol. Spectr..

[101] Rezaei S.D., Hayward J.A., Norden S., Pedersen J., Mills J., Hearps A.C., Tachedjian G. (2021). HERV-K Gag RNA and protein levels are elevated in malignant regions of the prostate in males with prostate cancer. Viruses.

[102] Hua T., Zeng Z., Chen J., Xue Y., Li Y., Sang Q. (2022). Human malignant rhabdoid tumor antigens as biomarkers and potential therapeutic targets. Cancers.

[103] Doucet-O’Hare T.T., DiSanza B.L., DeMarino C., Atkinson A.L., Rosenblum J.S., Henderson L.J., Johnson K.R., Kowalak J., Garcia-Montojo M., Allen S.J. (2021). SMARCB1 deletion in atypical teratoid rhabdoid tumors results in human endogenous retrovirus K (HML-2) expression. Sci. Rep..

[104] Sapkota S.S.H. (2022). Non-hodgkin lymphoma. StatPearls.

[105] GlaxoSmithKline, GileadSciences (2016). A Phase I/II Study of Safety and Efficacy of Lamivudine (EPIVIR®) and Tenofovir Disoproxil Fumarate (VIREAD®) Used to Lower the Plasma Level of Viral RNA of HERV-K(HML2) in Patients with Lymphoma. Identifier NCT01528865. University of Michigan Rogel Cancer Center. NCT01528865.

[106] Löwer J., Löwer R., Stegmann J., Frank H., Kurth R., Leukemia I.V., Neth R., Gallo R.C., Graf T., Mannweiler K., Winkler K. (1981). Retrovirus particle production in three of four human teratocarcinoma cell lines. Modern Trends in Human.

[107] Li M.D., Bronson D.L., Lemke T.D., Faras A.J. (1995). Restricted expression of New HERV-K members in human teratocarcinoma cells. Virology.

[108] Kassiotis G., Stoye J.P. (2016). Immune responses to endogenous retroelements: Taking the bad with the good. Nat. Rev. Immunol..

[109] Messerschmitt P.J., Garcia R.M., Abdul-Karim F.W., Greenfield E.M., Getty P.J. (2009). Osteosarcoma. JAAOS J. Am. Acad. Orthop. Surg..

[110] Moore D.D., Luu H.H., Peabody T.D., Attar S. (2014). Osteosarcoma. Cancer Trearment and Research. Orthopaedic Oncology; Primary and Metastatic Tumors of the Skeletal, System.

[111] Rodić N., Burns K.H. (2013). Long Interspersed Element–1 (LINE-1): Passenger or driver in human neoplasms?. PLoS Genet..

[112] Levine A.J., Ting D.T., Greenbaum B.D. (2016). P53 and the defenses against genome instability caused by transposons and repetitive elements. BioEssays.

[113] Wylie A., Jones A., D’Brot A., Lu W.-J., Kurtz P., Moran J., Rakheja D., Chen K., Hammer R., Comerford S. (2015). P53 genes function to restrain mobile elements. Genes. Dev..

[114] Clayton E.A., Wang L., Rishishwar L., Wang J., McDonald J.F., Jordan I.K. (2016). Patterns of transposable element expression and insertion in cancer. Front. Mol. Biosci..

[115] Dolci M., Favero C., Toumi W., Favi E., Tarantini L., Signorini L., Basile G., Bollati V., D’Alessandro S., Bagnoli P. (2020). Human endogenous retroviruses long terminal repeat methylation, transcription, and protein expression in human colon cancer. Front. Oncol..

[116] Mármol I., Sánchez-de-Diego C., Pradilla Dieste A., Cerrada E., Rodriguez Yoldi M.J. (2017). Colorectal carcinoma: A general overview and future perspectives in colorectal cancer. Int. J. Mol. Sci..

[117] Lin O.S., Verma M. (2009). Acquired risk factors for colorectal cancer. Cancer Epidemiology: Modifiable Factors.

[118] Mallo G.V., Fiedler F., Calvo E.L., Ortiz E.M., Vasseur S., Keim V., Morisset J., Iovanna J.L. (1997). Cloning and expression of the rat P8 CDNA, a new gene activated in pancreas during the acute phase of pancreatitis, pancreatic development, and regeneration, and which promotes cellular growth. J. Biol. Chem..

[119] Malicet C., Dagorn J.C., Neira J.L., Iovanna J.L. (2006). P8 and Prothymosin Alpha: Unity Is Strength. Cell Cycle.

[120] Malicet C., Giroux V., Vasseur S., Dagorn J.C., Neira J.L., Iovanna J.L. (2006). Regulation of apoptosis by the P8/prothymosin α complex. Proc. Natl. Acad. Sci. USA.

[121] Martin A.T., Li Xinyu A., Sanders J.A., Ye L., Frewer K., Hargest R., Jiang G.W. (2021). NUPR1 and its potential role in cancer and pathological conditions (review). Int. J. Oncol..

[122] Ko E.-J., Ock M.-S., Choi Y.-H., Iovanna J.L., Mun S., Han K., Kim H.-S., Cha H.-J. (2021). Human endogenous retrovirus (HERV)-K Env gene knockout affects tumorigenic characteristics of NUPR1 gene in DLD-1 colorectal cancer cells. Int. J. Mol. Sci..

[123] Gironella M., Malicet C., Cano C., Sandi M.J., Hamidi T., Tauil R.M.N., Baston M., Valaco P., Moreno S., Lopez F. (2009). P8/NUPR1 regulates DNA-repair activity after double-strand gamma irradiation-induced DNA damage. J. Cell. Physiol..

[124] Hamidi T., Algül H., Cano C.E., Sandi M.J., Molejon M.I., Riemann M., Calvo E.L., Lomberk G., Dagorn J.-C., Weih F. (2012). Nuclear protein 1 promotes pancreatic cancer development and protects cells from stress by inhibiting apoptosis. J. Clin. Investig..

[125] Encinar J.A., Mallo G.V., Mizyrycki C., Giono L., González-Ros J.M., Rico M., Cánepa E., Moreno S., Neira J.L., Iovanna J.L. (2001). Human P8 is a HMG-I/Y-like protein with DNA binding activity enhanced by phosphorylation. J. Biol. Chem..

[126] Grasso D., Garcia M.N., Hamidi T., Cano C., Calvo E., Lomberk G., Urrutia R., Iovanna J.L. (2014). Genetic inactivation of the pancreatitis-inducible gene Nupr1 Impairs PanIN formation by modulating KrasG12D-induced senescence. Cell Death Differ..

[127] Ashktorab H., Daremipouran M., Goel A., Varma S., Leavitt R., Sun X., Brim H. (2014). DNA methylome profiling identifies novel methylated genes in African American patients with colorectal neoplasia. Epigenetics.

[128] Gualtieri A., Andreola F., Sciamanna I., Sinibaldi Vallebona P., Serafino A., Spadafora C. (2013). Increased expression and copy number amplification of LINE-1 and SINE B1 retrotransposable elements in murine mammary carcinoma progression. Oncotarget.

[129] Chappell G., Kutanzi K., Uehara T., Tryndyak V., Hong H.-H., Hoenerhoff M., Beland F.A., Rusyn I., Pogribny I.P. (2014). Genetic and epigenetic changes in fibrosis-associated hepatocarcinogenesis in mice. Int. J. Cancer.

[130] DeRoo L.A., Bolick S.C.E., Xu Z., Umbach D.M., Shore D., Weinberg C.R., Sandler D.P., Taylor J.A. (2014). Global DNA methylation and one-carbon metabolism gene polymorphisms and the risk of breast cancer in the sister study. Carcinogenesis.

[131] Ogino S., Nishihara R., Lochhead P., Imamura Y., Kuchiba A., Morikawa T., Yamauchi M., Liao X., Qian Z.R., Sun R. (2013). Prospective study of family history and colorectal cancer risk by tumor LINE-1 methylation level. JNCI J. Natl. Cancer Inst..

[132] Rhyu D.-W., Kang Y.-J., Ock M.-S., Eo J.-W., Choi Y.-H., Kim W.-J., Leem S.-H., Yi J.-M., Kim H.-S., Cha H.-J. (2014). Expression of human endogenous retrovirus Env genes in the blood of breast cancer patients. Int. J. Mol. Sci..

[133] Liang Q., Ding J., Xu R., Xu Z., Zheng S. (2009). Identification of a novel human endogenous retrovirus and promoter activity of its 5′ U3. Biochem. Biophys. Res. Commun..

[134] Zare M., Mostafaei S., Ahmadi A., Azimzadeh Jamalkandi S., Abedini A., Esfahani-Monfared Z., Dorostkar R., Saadati M. (2018). Human endogenous retrovirus Env genes: Potential blood biomarkers in lung cancer. Microb. Pathog..

[135] Zhang M., Liang J.Q., Zheng S. (2019). Expressional activation and functional roles of human endogenous retroviruses in cancers. Rev. Med. Virol..

[136] Manghera M., Ferguson J., Douville R. (2015). HERV-K polyprotein processing and reverse transcriptase expression in human cell line models of neurological disease. Viruses.

[137] Harbeck N., Penault-Llorca F., Cortes J., Gnant M., Houssami N., Poortmans P., Ruddy K., Tsang J., Cardoso F. (2019). Breast cancer. Nat. Rev. Dis. Prim..

[138] Golan M., Hizi A., Resau J.H., Yaal-Hahoshen N., Reichman H., Keydar I., Tsarfaty I. (2008). Human endogenous retrovirus (HERV-K) reverse transcriptase as a breast cancer prognostic marker. Neoplasia.

[139] Ejthadi H.D., Martin J.H., Junying J., Roden D.A., Lahiri M., Warren P., Murray P.G., Nelson P.N. (2005). A novel multiplex RT-PCR system detects human endogenous retrovirus-K in breast cancer. Arch. Virol..

[140] Wang-Johanning F., Frost A., Johanning G., Khazaeli M., LoBuglio A., Shaw D., Strong T. (2001). Expression of human endogenous retrovirus k envelope transcripts in human breast cancer. Clin. Cancer Res..

[141] Wang-Johanning F., Li M., Esteva F.J., Hess K.R., Yin B., Rycaj K., Plummer J.B., Garza J.G., Ambs S., Johanning G.L. (2014). Human endogenous retrovirus type K antibodies and mRNA as serum biomarkers of early-stage breast cancer. Int. J. Cancer.

[142] Wang-Johanning F., Radvanyi L., Rycaj K., Plummer J.B., Yan P., Sastry K.J., Piyathilake C.J., Hunt K.K., Johanning G.L. (2008). Human endogenous retrovirus K triggers an antigen-specific immune response in breast cancer patients. Cancer Res..

[143] Zhao J., Rycaj K., Geng S., Li M., Plummer J.B., Yin B., Liu H., Xu X., Zhang Y., Yan Y. (2011). Expression of human endogenous retrovirus type K envelope protein is a novel candidate prognostic marker for human breast cancer. Genes Cancer.

[144] Wang-Johanning F., Rycaj K., Plummer J.B., Li M., Yin B., Frerich K., Garza J.G., Shen J., Lin K., Yan P. (2012). Immunotherapeutic potential of anti-human endogenous retrovirus-K envelope protein antibodies in targeting breast tumors. JNCI J. Natl. Cancer Inst..

[145] Rosen R., Sapra A. (2022). TNM classification. StatPearls.

[146] Rivlin N., Brosh R., Oren M., Rotter V. (2011). Mutations in the P53 tumor suppressor gene: Important milestones at the various steps of tumorigenesis. Genes Cancer.

[147] Reis B., Jungbluth A., Frosina D., Holz M., Ritter E., Nakayama E., Ishida T., Obata Y., Carver B., Scher H. (2013). Prostate cancer progression correlates with increased humoral immune response to a human rndogenous retrovirus Gag protein. Clin. Cancer Res..

[148] Hu J., Han J., Li H., Zhang X., Liu L., Chen F., Zeng B. (2018). Human embryonic kidney 293 cells: A vehicle for biopharmaceutical manufacturing, structural biology, and electrophysiology. Cells Tissues Organs.

[149] Wegener M., Müller-McNicoll M. (2018). Nuclear retention of mRNAs—quality control, gene regulation and human disease. Semin. Cell. Dev. Biol..

[150] Löwer R., Tönjes R.R., Korbmacher C., Kurth R., Löwer J. (1995). Identification of a Rev-related protein by analysis of spliced transcripts of the human endogenous retroviruses HTDV/HERV-K. J. Virol..

[151] Yang J., Bogerd H.P., Peng S., Wiegand H., Truant R., Cullen B.R. (1999). An ancient family of human endogenous retroviruses encodes a functional homolog of the HIV-1 Rev protein. Proc. Natl. Acad. Sci. USA.

[152] Zeng T., Fedeli M.A., Tanda F., Wang Y., Yang D., Xue B., Jia L., Palmieri G., Sechi L.A., Kelvin D.J. (2021). Whole-exome sequencing of prostate cancer in sardinian identify recurrent UDP-glucuronosyltransferase amplifications. J. Cancer.

[153] Wang Z., Wang Y., Zhang J., Hu Q., Zhi F., Zhang S., Mao D., Zhang Y., Liang H. (2017). Significance of the TMPRSS2:ERG gene fusion in prostate cancer. Mol. Med. Rep..

[154] Jia L., Liu M., Yang C., Li H., Liu Y., Han J., Zhai X., Wang X., Li T., Li J. (2022). Comprehensive identification and characterization of the HERV-K (HML-9) group in the human genome. Retrovirology.

[155] Sizemore G.M., Pitarresi J.R., Balakrishnan S., Ostrowski M.C. (2017). The ETS family of oncogenic transcription factors in solid tumours. Nat. Rev. Cancer.

[156] Stine Z.E., Walton Z.E., Altman B.J., Hsieh A.L., Dang C.V. (2015). MYC, metabolism, and cancer. Cancer Discov..

[157] Kraus B., Fischer K., Sliva K., Schnierle B.S. (2014). Vaccination directed against the human endogenous retrovirus-K (HERV-K) Gag protein slows HERV-K Gag expressing cell growth in a murine model system. Virol. J..

[158] Ragonnaud E., Neukirch L., Pedersen I., Daradoumis J., Daradoumis J., Grunddal K., Duvnjak L., Bermejo A., Schroedel S., Thirion C. (2022). P03.03 Active immunization against human endogenous retrovirus type K (HERV-K) as an immunotherapeutic strategy against solid tumors. J. Immunother. Cancer.

[159] Wang-Johanning F., Rycaj K., Huang M., Plummer J., Marks J., Johanning G., Rosenblum M. (2007). Anti-HERV-K antibody 6H5 and 6H5/RGel inhibit cell growth and induce apoptosis in breast and ovarian cancer cells. Cancer Res..

[160] Perron H., Medina J. (2018). Anti-HERV-K Envelope Antibody and Uses Thereof. https://patents.google.com/patent/EP3351265A1/en.

[161] Karimi A., Sheervalilou R., Kahroba H. (2019). A new insight on activation of human endogenous retroviruses (HERVs) in malignant melanoma upon exposure to CuSO4. Biol. Trace Elem. Res..

[162] Carlini F., Ridolfi B., Molinari A., Parisi C., Bozzuto G., Toccacieli L., Formisano G., De Orsi D., Paradisi S., Grober O.M.V. (2010). The reverse transcription inhibitor abacavir shows anticancer activity in prostate cancer cell lines. PLoS ONE.

[163] Ko E.J., Kim E.T., Kim H., Lee C.M., Koh S.B., Eo W.K., Kim H., Oh Y.L., Ock M.S., Kim K.H. (2022). Effect of human endogenous retrovirus-K Env gene knockout on proliferation of ovarian cancer cells. Genes. Genom..

[164] Temerozo J.R., Fintelman-Rodrigues N., dos Santos M.C., Hottz E.D., Sacramento C.Q., de Paula Dias da Silva A., Mandacaru S.C., dos Santos Moraes E.C., Trugilho M.R.O., Gesto J.S.M. (2022). Human endogenous retrovirus K in the respiratory tract is associated with COVID-19 physiopathology. Microbiome.

[165] Hashemian S.M., Farhadi T., Velayati A.A. (2020). A review on remdesivir: A possible promising agent for the treatment of COVID-19. Drug. Des. Devel. Ther..

[166] Sacramento C.Q., Fintelman-Rodrigues N., Temerozo J.R., de Paula Dias Da Silva A., de Paula Dias Da Silva A., da Silva C.D.S., Ferreira A.C., Mattos M., Pão C.R.R., de Freitas C.S. (2021). In vitro antiviral activity of the anti-HCV drugs daclatasvir and sofosbuvir against SARS-CoV-2, the aetiological agent of COVID-19. J. Antimicrob. Chemother..

[167] Li P., Zheng Y., Chen X. (2017). Drugs for autoimmune inflammatory diseases: From small molecule compounds to anti-TNF biologics. Front. Pharmacol..

[168] Moru X., Kun Q., Hongxia S., Yongxiu Y., Venugopal N., Jianqiang Y., Aijian Q. (2022). Glycosylation of ALV-J envelope protein at sites 17 and 193 is pivotal in the virus infection. J. Virol..

[169] Bermejo A., Daradoumis J., Azcoaga P., Ragonnaud E., Neukrich L., Nielsen K., Andersoon A., Scroedel S., Thirion C., Ørskov C. (2022). P03.05 vaccine immunotherapy against human endogenous retrovirus: A focus on anti-HERV-K antibodies. J. Immunother. Cancer.

[170] Harris E.D. (2001). Copper Homeostasis: The role of cellular transporters. Nutr. Rev..

[171] Tapiero H., Townsend D.M., Tew K.D. (2003). Trace elements in human physiology and pathology. Copper. Biomed. Pharmacother..

[172] Osredkar J., Sustar N. (2011). Copper and zinc, biological role and significance of copper/zinc imbalance. J. Clin. Toxicol..

[173] D’Mello S.A.N., Finlay G.J., Baguley B.C., Askarian-Amiri M.E. (2016). Signaling pathways in melanogenesis. Int. J. Mol. Sci..

[174] Grubman A., White A.R. (2014). Copper as a key regulator of cell signalling pathways. Expert. Rev. Mol. Med..

[175] Arigony A.L.V., de Oliveira I.M., Machado M., Bordin D.L., Bergter L., Prá D., Pêgas Henriques J.A. (2013). The influence of micronutrients in cell culture: A reflection on viability and genomic stability. Biomed. Res. Int..

[176] Ibba G., Piu C., Uleri E., Serra C., Dolei A. (2018). Disruption by SaCas9 endonuclease of HERV-K Env, a retroviral gene with oncogenic and neuropathogenic potential, inhibits molecules involved in cancer and amyotrophic lateral sclerosis. Viruses.

[177] Brezgin S., Kostyusheva A., Kostyushev D., Chulanov V. (2019). Dead Cas systems: Types, principles, and applications. Int. J. Mol. Sci..

[178] Nath A. (2022). HERV-K Suppression Using Antiretroviral Therapy in Volunteers with Amyotrophic Lateral Sclerosis (ALS). Identifier NCT02437110. National Institute of Neurological Disorders and Stroke. NCT02437110.

[179] Olasupo O., Lowe M., Krishan A., Collins P., Iorio A., Matino D. (2021). Clotting factor concentrates for preventing bleeding and bleeding-related complications in previously treated individuals with haemophilia A or B. Cochrane Database Syst. Rev..

[180] Gabay C., Lamacchia C., Palmer G. (2010). IL-1 Pathways in inflammation and human diseases. Nat. Rev. Rheumatol..

[181] Zhao R., Chinai J.M., Buhl S., Scandiuzzi L., Ray A., Jeon H., Ohaegbulam K.C., Ghosh K., Zhao A., Scharff M.D. (2013). HHLA2 is a member of the B7 family and inhibits human CD4 and CD8 T-cell function. Proc. Natl. Acad. Sci. USA.

[182] Amatya N., Garg A.V., Gaffen S.L. (2017). IL-17 signaling: The yin and the yang. Trends Immunol..

[183] Wang X., Zhao C., Zhang C., Mei X., Song J., Sun Y., Wu Z., Shi W. (2019). Increased HERV-E Clone 4–1 expression contributes to DNA hypomethylation and IL-17 release from CD4+ T Cells via MiR-302d/MBD2 in systemic lupus erythematosus. Cell Commun. Signal..

[184] Dorward D.A., Russell C.D., Um I.H., Elshani M., Armstrong S.D., Penrice-Randal R., Millar T., Lerpiniere C.E.B., Tagliavini G., Hartley C.S. (2020). Tissue-specific immunopathology in fatal COVID-19. Am. J. Respir. Crit. Care Med..

[185] Merad M., Martin J.C. (2020). Pathological inflammation in patients with COVID-19: A key role for monocytes and macrophages. Nat. Rev. Immunol..

[186] Ferreira A.C., Soares V.C., de Azevedo-Quintanilha I.G., da Silva Gomes Dias S., Fintelman-Rodrigues N., Sacramento C.Q., Mattos M., de Freitas C.S., Temerozo J.R., Teixeira L. (2021). SARS-CoV-2 engages inflammasome and pyroptosis in human primary monocytes. Cell Death Discov..

[187] Rodrigues T.S., de Sá K.S.G., Ishimoto A.Y., Becerra A., Oliveira S., Almeida L., Gonçalves A.V., Perucello D.B., Andrade W.A., Castro R. (2020). Inflammasomes are activated in response to SARS-CoV-2 infection and are associated with COVID-19 severity in patients. J. Exp. Med..

[188] Tay M.Z., Poh C.M., Rénia L., MacAry P.A., Ng L.F.P. (2020). The trinity of COVID-19: Immunity, inflammation and intervention. Nat. Rev. Immunol..

